# Large-Scale Analysis of Kinase Signaling in Yeast Pseudohyphal Development Identifies Regulation of Ribonucleoprotein Granules

**DOI:** 10.1371/journal.pgen.1005564

**Published:** 2015-10-08

**Authors:** Christian A. Shively, Hye Kyong Kweon, Kaitlyn L. Norman, Dattatreya Mellacheruvu, Tao Xu, Daniel T. Sheidy, Craig J. Dobry, Ivan Sabath, Eric E. P. Cosky, Elizabeth J. Tran, Alexey Nesvizhskii, Philip C. Andrews, Anuj Kumar

**Affiliations:** 1 Program in Cellular and Molecular Biology, University of Michigan, Ann Arbor, Michigan, United States of America; 2 Department of Molecular, Cellular, and Developmental Biology, University of Michigan, Ann Arbor, Michigan, United States of America; 3 Department of Biological Chemistry, University of Michigan Medical School, Ann Arbor, Michigan, United States of America; 4 Department of Pathology, University of Michigan Medical School, Ann Arbor, Michigan, United States of America; 5 Department of Computational Medicine and Bioinformatics, University of Michigan Medical School, Ann Arbor, Michigan, United States of America; 6 Department of Biochemistry, Purdue University, West Lafayette, Indiana, United States of America; SUNY-Buffalo, UNITED STATES

## Abstract

Yeast pseudohyphal filamentation is a stress-responsive growth transition relevant to processes required for virulence in pathogenic fungi. Pseudohyphal growth is controlled through a regulatory network encompassing conserved MAPK (Ste20p, Ste11p, Ste7p, Kss1p, and Fus3p), protein kinase A (Tpk2p), Elm1p, and Snf1p kinase pathways; however, the scope of these pathways is not fully understood. Here, we implemented quantitative phosphoproteomics to identify each of these signaling networks, generating a kinase-dead mutant in filamentous *S*. *cerevisiae* and surveying for differential phosphorylation. By this approach, we identified 439 phosphoproteins dependent upon pseudohyphal growth kinases. We report novel phosphorylation sites in 543 peptides, including phosphorylated residues in Ras2p and Flo8p required for wild-type filamentous growth. Phosphoproteins in these kinase signaling networks were enriched for ribonucleoprotein (RNP) granule components, and we observe co-localization of Kss1p, Fus3p, Ste20p, and Tpk2p with the RNP component Igo1p. These kinases localize in puncta with GFP-visualized mRNA, and *KSS1* is required for wild-type levels of mRNA localization in RNPs. Kss1p pathway activity is reduced in *lsm1*Δ/Δ and *pat1*Δ/Δ strains, and these genes encoding P-body proteins are epistatic to *STE7*. The P-body protein Dhh1p is also required for hyphal development in *Candida albicans*. Collectively, this study presents a wealth of data identifying the yeast phosphoproteome in pseudohyphal growth and regulatory interrelationships between pseudohyphal growth kinases and RNPs.

## Author Summary

Eukaryotic cells affect precise changes in shape and growth in response to environmental and nutritional stress, enabling cell survival and wild-type function. The single-celled budding yeast provides a striking example, undergoing a set of changes under conditions of nitrogen or glucose limitation resulting in the formation of extended cellular chains or filaments. Related filamentous growth transitions are required for virulence in pathogenic fungi and have been studied extensively; however, the full scope of signaling underlying the filamentous growth transition remains to be determined. Here, we used a combination of genetics and proteomics to identify proteins that undergo phosphorylation dependent upon kinases required for filamentous growth. Within this protein set, we identified novel sites of phosphorylation in the yeast proteome and extensive phosphorylation of mRNA-protein complexes regulating mRNA decay and translation. The data indicate an interrelationship between filamentous growth and these ubiquitously conserved sites of RNA regulation: the RNA-protein complexes are required for the filamentous growth transition, and a well studied filamentous growth signaling kinase is required for wild-type numbers of RNA-protein complexes. This interdependence is previously unappreciated, highlighting an additional level of translational control underlying this complex growth transition.

## Introduction

The pseudohyphal growth response is a complex morphogenetic program in which fungal cells transition from a yeast-like growth form to a filamentous state, with cells remaining physically connected after cytokinesis in elongated structures [1–3]. This growth transition is evident in several strains of *S*. *cerevisiae* (e.g., Σ1278b and SK1) [4, 5] and is triggered by numerous conditions, including nitrogen limitation, glucose limitation, the presence of starch as a sole carbon source, and elevated levels of fusel alcohols [1, 6–9]. Since yeast pseudohyphal growth is principally induced in response to nutrient stress, it is widely presumed to be a nutritional foraging mechanism [10]. Pseudohyphal growth has been studied intensely in *S*. *cerevisiae* as an informative model of related processes of filamentous growth evident in many fungi. In particular, the pseudohyphal growth transition in *S*. *cerevisiae* is closely related to filamentous growth transitions enabling the formation of pseudohyphae and true hyphae with parallel-sided cell walls in the opportunistic human fungal pathogen *Candida albicans* [11–13]. Further, the ability to form hyphae and to transition between these growth forms is required for virulence in *C*. *albicans* [14–16].

The molecular basis of yeast pseudohyphal growth is extensive. Pseudohyphal formation in *S*. *cerevisiae* is enabled by changes in cell polarity, cytoskeletal organization, and cell adhesion controlled through a regulatory network encompassing a core set of strongly conserved signaling modules [17–20]. Yeast cells contain several mitogen-activated protein kinase (MAPK) pathways, and elegant studies in the mid-1990s identified the cascade of Ste11p, Ste7p, and Kss1p as a pseudohyphal growth activator [21–23]. Within this pseudohyphal growth MAPK pathway, the upstream p21-activated kinase Ste20p phosphorylates and activates Ste11p, and this phosphorylation signal is propagated through Kss1p to the heterodimeric transcription factor Ste12p/Tec1p [24, 25]. Ste11p and Ste7p are also components of a pheromone-responsive MAPK cascade containing the MAPK Fus3p [26, 27]. Fus3p negatively regulates pseudohyphal growth by phosphorylating Tec1p Thr273, targeting Tec1p for degradation [28].

In addition to these MAPK pathways, cAMP-dependent protein kinase A (PKA) is a key regulator of pseudohyphal development. In *S*. *cerevisiae*, PKA consists of the Bcy1p regulatory subunit and one of three catalytic subunits, Tpk1p, Tpk2p, and Tpk3p [29, 30]. Tpk2p phosphorylates the filamentous growth transcriptional activator Flo8p, and deletion of *TPK2* reduces pseudohyphal growth [31, 32]. The AMP-activated kinase Snf1p is a well-studied transcription factor required for derepressed expression of glucose-repressible genes [33]. Snf1p represses the pseudohyphal growth negative regulators Nrg1p and Nrg2p, resulting in transcriptional activation of *FLO11*, among other targets [34]. *FLO11* encodes a GPI-anchored cell surface flocculin required for pseudohyphal growth, acting as a downstream effector of the Kss1p MAPK pathway through Ste12p/Tec1p, the PKA pathway through Flo8p, and Snf1p as described [35, 36]. Snf1p Thr210 is phosphorylated by Elm1p, which regulates cellular morphogenesis and cytokinesis [37].

The core components of these signaling pathways are well established, but the set of targets of each signaling module are not as clearly defined with respect to the gene network contributing to pseudohyphal growth. Systematic analysis of loss-of-function mutants revealed that approximately 700 genes are required for wild-type pseudohyphal growth [38, 39], and a partially overlapping set of 551 genes promotes invasive growth upon galactose-induced overexpression [40]. In particular, the regulation of stress-responsive processes during pseudohyphal growth is a point of ongoing study, indicating counterbalanced control of autophagy through Tor/PKA [41] and an extensive glucose-regulated signaling network encompassing Snf1p and related pathways [34, 42]. Pseudohyphal growth gene networks have been analyzed globally for regulatory control at the level of transcription [43, 44], but kinase signaling networks regulating filamentous growth have been constructed predominantly from individual studies of a given kinase and target. Although kinase signaling in yeast has been analyzed effectively through mass spectrometry-based phosphoproteomics [45–48], these methods had not been applied to define kinase networks controlling pseudohyphal growth in a filamentous strain of *S*. *cerevisiae*. Here, we implemented quantitative phosphoproteomics to identify signaling networks for a set of kinases that regulate filamentation, with the results revealing a wealth of previously unknown phosphorylation sites, phosphorylated residues in Ras2p and Flo8p required for pseudohyphal growth, and MAPK regulation of ribonucleoprotein complexes via the Kss1p cascade.

## Results

### Identifying pseudohyphal growth kinase signaling networks by quantitative phosphoproteomics

To dissect kinase signaling networks regulating yeast pseudohyphal growth, we adopted a straightforward approach, generating a loss-of-function mutation in relevant pseudohyphal growth kinases and surveying the resulting changes in phosphorylation. For this study, we constructed catalytically impaired kinase-dead alleles in the filamentous Σ1278b strain of *S*. *cerevisiae* for each of the following kinases: Ste20p, Ste11p, Ste7p, Kss1p, Fus3p, Tpk2p, Elm1p, and Snf1p. The signaling context of each protein is indicated in S1A Fig. Mutant kinase alleles were generated by deletion of each kinase gene and introduction of a low-copy centromeric plasmid bearing the native gene promoter and mutated coding sequence encoding a Lys-to-Arg substitution at the conserved residue in the catalytic loop of each respective kinase domain. Resulting filamentous growth phenotypes are presented in S1 Table; images of these kinase-dead mutants as well as background deletion strains and isogenic strains carrying wild-type kinase genes are shown in S1B–S1D Fig.

**Fig 1:**
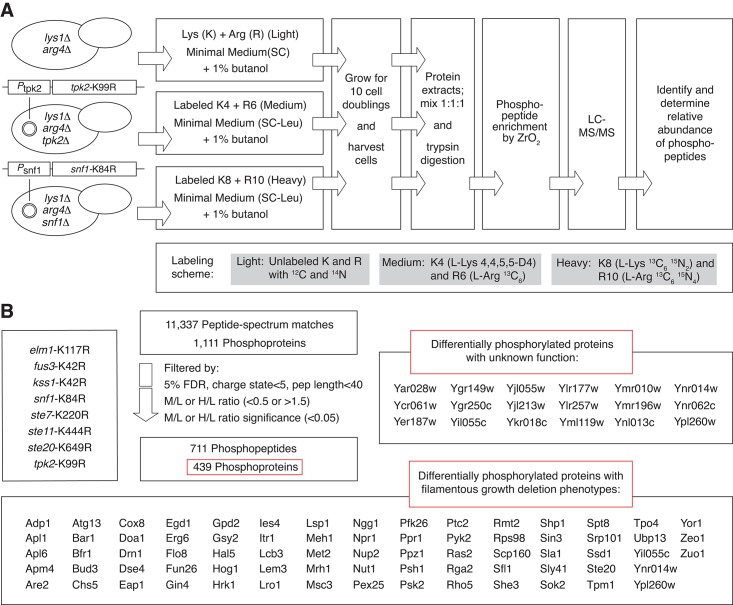
Quantitative phosphoproteomic analysis of pseudohyphal growth kinase signaling in a filamentous strain of *S*. *cerevisiae* by SILAC. A) The diagram presents an overview of the major steps in protein labeling and mass spectrometry-based identification of kinase-dependent phosphorylation. The labeling scheme for a triplex experiment including strains with kinase-dead *tpk2-*K99R and *snf1-*K84R alleles is indicated as an example of the full study. B) Summary of mass spectrometry results.

Differential phosphorylation in the kinase-dead mutants relative to wild type was assessed by quantitative phosphoproteomics using stable isotopic labeling of amino acids in cell culture (SILAC) [49]. To implement this SILAC-based approach, the wild type and kinase mutant strains in Σ1278b were made auxotrophic for lysine and arginine by deletion of *LYS1* and *ARG4*. The resulting strains were cultured in triplicate under conditions inducing pseudohyphal growth in medium containing either isotopically labeled or unlabeled lysine and arginine. Protein extracts from the cultures were enriched for phosphopeptides, and the enriched fractions were analyzed by liquid chromatography coupled with tandem mass spectrometry to determine the identity and relative abundance of the phosphopeptides. This experimental design and workflow is summarized in Fig 1A.

### An extensive phosphorylation network dependent upon pseudohyphal growth kinase activity

By SILAC-based phosphoproteomics, we identified 11,337 peptide-to-spectrum matches and filtered the peptides by criteria indicated in Experimental Procedures. The resulting data indicate 3,699 unique phosphopeptides, corresponding to 1,111 proteins. In total, we observed 711 peptides that exhibited a change in phosphorylation (SILAC ratio ≥1.5 or ≤0.5 with statistical significance ≤0.05) in the respective kinase mutants relative to wild type (Fig 1B). This differentially phosphorylated peptide set corresponds to 439 phosphoproteins. A full listing of these data is provided in S2 Table. In addition to Ser/Thr phosphorylation, we also identify 137 peptides with a phosphorylated tyrosine residue, corresponding to 110 yeast proteins. By our experimental design, the proteins identified in this study encompass both direct and indirect targets of the respective pseudohyphal growth kinases. These phosphoproteins encompass eighteen functionally uncharacterized proteins and 73 proteins whose corresponding genes yield pseudohyphal growth phenotypes upon deletion (Fig 1B) [38, 39].

### Previously unidentified phosphorylation sites in the proteome of the filamentous Σ1278b strain

**Fig 2:**
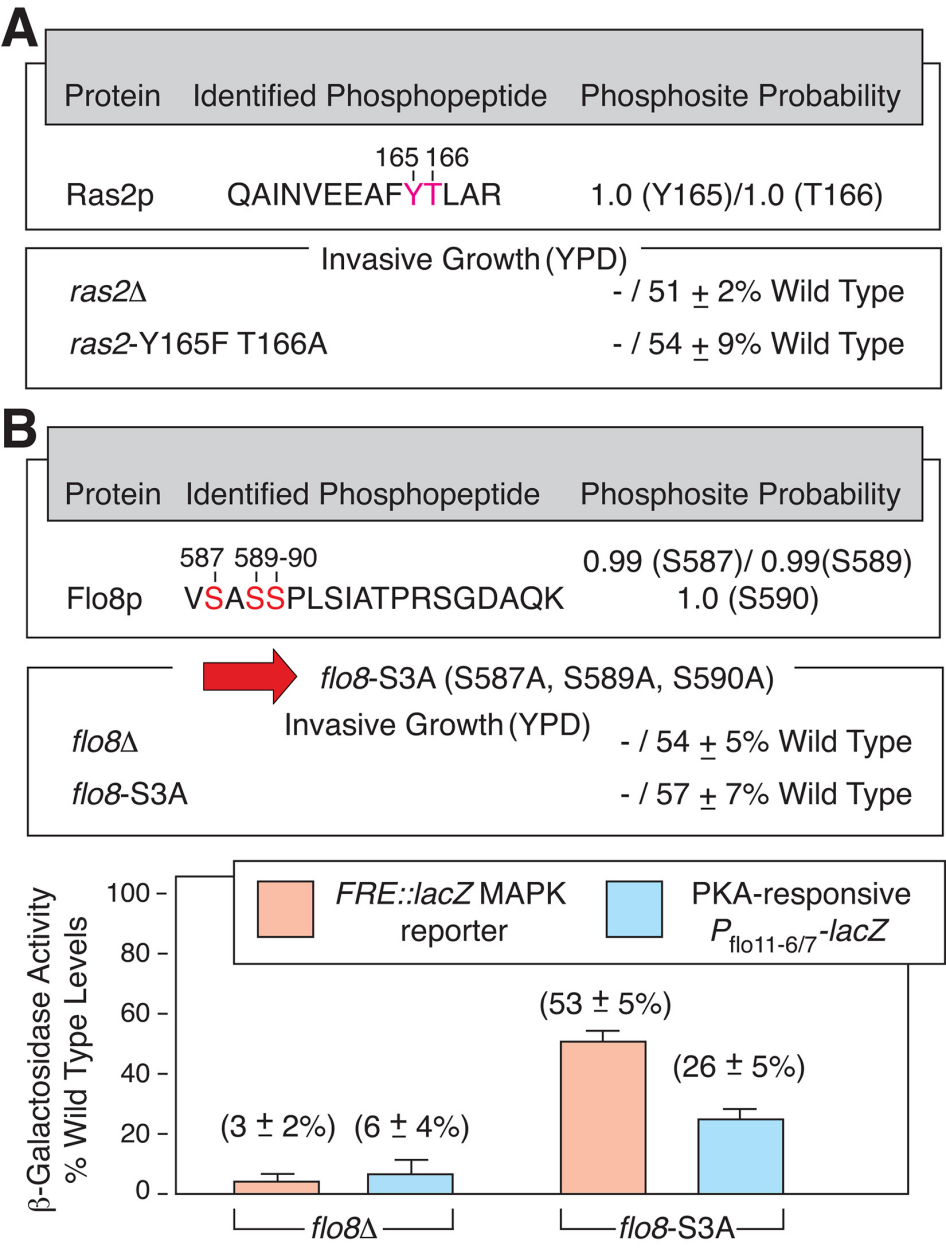
Newly identified phosphorylation sites in Ras2p and Flo8p are required for wild-type yeast invasive growth. A) Previously unreported phosphorylation sites in Ras2p are shown in red with indicated probability that the sites were correctly identified. Site-directed mutagenesis was used to generate an integrated allele of *ras2* encoding Phe and Ala substitutions at Y165 and T166, respectively. The mutant exhibits diminished invasive growth (“-”) on YPD medium. Invasive growth was scored quantitatively as mean pixel intensity of the spotted area post-washing relative to the mean pixel intensity before washing. Error bars indicate the standard deviation from three independent trials. B) Novel phosphorylation sites in Flo8p are shown in red. A mutant encoding alanine at each residue (*flo8-*S3A) yields decreased invasive growth and decreased transcriptional activity of *lacZ* reporters responsive to Ste12/Tec1p regulation (downstream of the Kss1p MAPK pathway, pFRE-*lacZ*) and Flo8p-binding (PKA-regulated, *P*_flo11-6/7_), respectively.

These mass spectrometry studies provide a substantial catalog of previously unreported phosphorylation sites across the yeast proteome. Due to the lack of a centralized repository of previously identified phosphopeptides, novel sites were identified through a multi-step process: we first generated a compendium of known phosphorylation sites culled from phosphopeptide databases (Materials and Methods), and we subsequently mapped peptides and phosphorylation sites from each of these databases onto the yeast proteome along with phosphosites identified in our data. Allowing for inherent uncertainties in both our data and reported phosphorylation sites from the community databases, we identified sites that were well distinct from those previously reported. A listing of potentially novel phosphorylation sites can be accessed from S2 Table. Interestingly, as indicated in Fig 2, we identified previously unreported phosphorylation sites in the GTP-binding protein Ras2p (Y165, T166) and the pseudohyphal growth transcription factor Flo8p (S587, S589, S590), both proteins being required for filamentous growth [50, 51]. Mutation of these sites to encode non-phosphorylatable residues results in decreased invasive growth (S2 Fig). A *flo8* mutant encoding alanine at residues 587, 589, and 590 (*flo8*-S3A) results in decreased production of *lacZ* driven from a segment of the *FLO11* promoter containing Flo8p-binding sites (*P*_flo11-6/7_). Additionally, the *flo8*-S3A allele yields decreased activity of a *lacZ* reporter driven by a filamentation-responsive element (FRE) recognized by the Kss1p-regulated Ste12p/Tec1p transcription factor [22] (Fig 2B). The *ras2*-Y165F/T166A and *flo8*-S3A alleles encode proteins that can be visualized as GFP chimeras exhibiting wild-type localization to the plasma membrane and nucleus, respectively (S2 Fig).

### The pseudohyphal growth kinase signaling network is enriched for proteins in RNP granules

**Fig 3:**
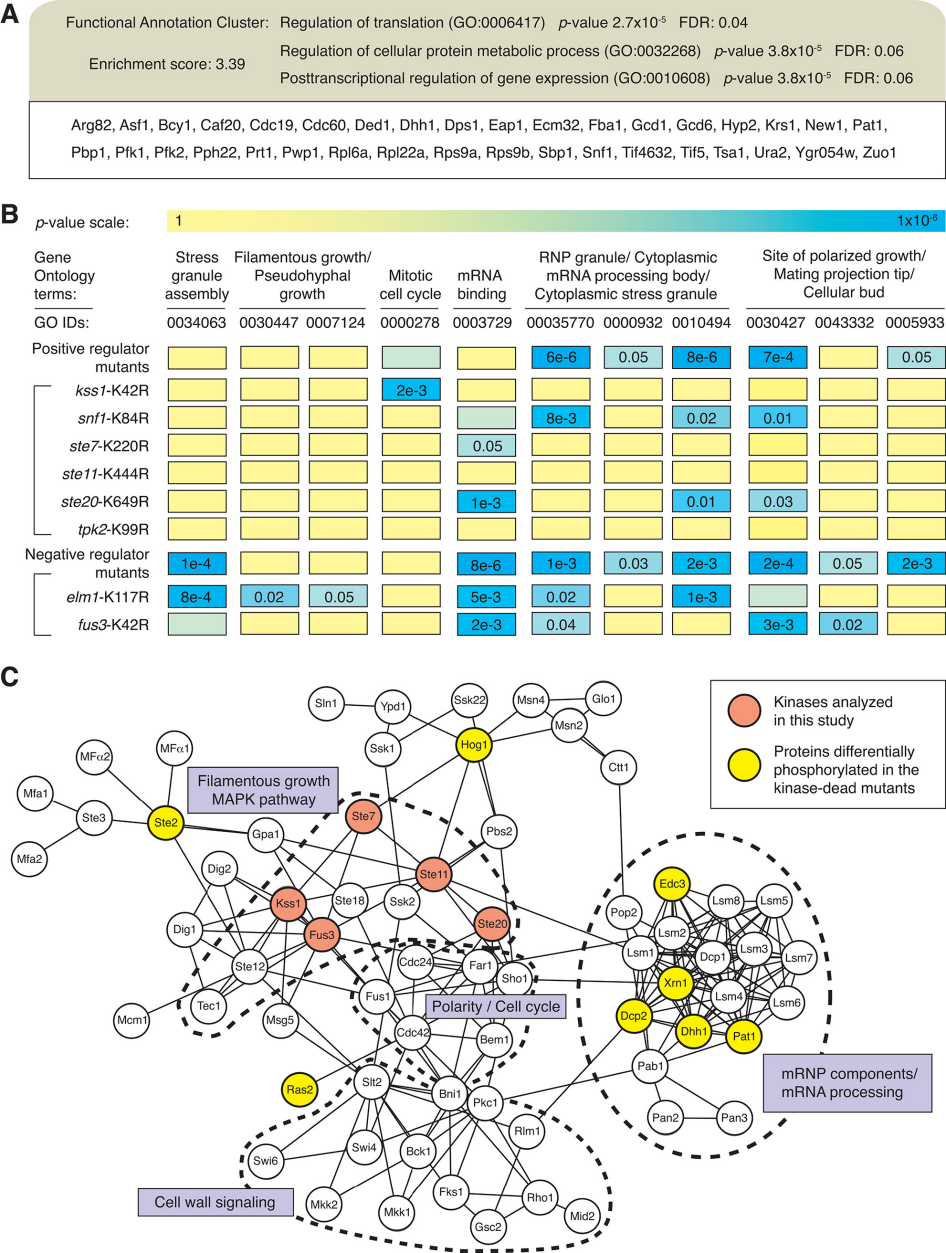
The yeast pseudohyphal growth kinase signaling network is enriched in proteins contributing to mRNP granule function. A) Analysis of phosphoproteins identified as being dependent upon yeast pseudohyphal growth kinases indicates a cluster of proteins involved in translational regulation, encompassing protein components of RNPs. B) The diagram presents a heat map for the enrichment of indicated GO terms (columns) in the respective mass spectrometry data sets generated from the kinase mutants (rows). For convenience, GO IDs are listed below each GO term. Kinases are grouped as positive and negative regulators and presented as individual data sets. C) Connectivity map of proteins in KEGG pathways associated with pseudohyphal growth (MAPK signaling pathway sce04011, cell cycle sce04111, meiosis sce04113) and proteins identified as being components of either P-bodies or stress granules. Lines indicate interactions annotated in KEGG and iRefIndex. Dashed lines identify clusters of proteins involved in the indicated processes.

To identify cellular processes involved in the pseudohyphal growth transition, we mined the collective set of proteins differentially phosphorylated in the kinase-dead mutants for statistically significant enrichment of associated Gene Ontology (GO) terms using the Biological Process, Molecular Function, and Cellular Component vocabularies. In addition to the expected identification of terms associated with pseudohyphal growth and polarized growth, this analysis indicated enrichment for proteins involved in translational regulation (Fig 3A). Using the DAVID bioinformatics suite, we identified a cluster of genes annotated with related GO terms involving the regulation of translation (GO ID:0006417), the regulation of cellular protein metabolic process (GO ID:0032268), and the posttranscriptional regulation of gene expression (GO ID:0010608). The gene set contains protein components of mRNA-protein granules, and gene sets annotated with the GO terms RNP granule (GO ID:00035770), cytoplasmic mRNA processing body (GO ID:0000932), and cytoplasmic stress granule (GO ID:0010494) are statistically enriched in the set of differentially phosphorylated proteins observed for many of the kinase-dead mutants (Fig 3B). It is noteworthy that the subset of identified proteins exhibiting increased phosphorylation in the kinase-dead strains, presumably encompassing indirect kinase targets, is not enriched for GO terms associated with RNP granules. A listing of GO terms enriched in this hyper-phosphorylated protein subset is presented in S3 Table.

Cytoplasmic stress granules and mRNA processing bodies (P-bodies) are two classes of RNPs observed in yeast, with compositional and presumed functional similarities to large families of RNP particles observed throughout eukaryotes. As reviewed in Buchan and Parker [52], the stress-induced RNPs contain non-translating mRNAs and function in an mRNP cycle, wherein mRNA may traffic between mRNPs that exhibit a dynamic protein makeup. Classically, P-bodies are thought to be aggregates of mRNA with proteins involved in translational repression, deadenylation, decapping, and 5’-to-3’ exonucleolytic mRNA decay [53, 54], while the protein composition of yeast stress granules encompasses translation initiation factors suggestive of associated RNAs stalled in translation initiation [55, 56]. Our phosphoproteomic analysis identifies pseudohyphal growth (PHG) kinase-dependent phosphorylation of proteins localized in P-bodies (Dcp2p, Ded1p, Dhh1p, Edc3p, Pat1p, Sbp1p, and Xrn1p) and stress granules (Eap1p, Hrp1p, Pbp1p, and Ygr250cp), as well as proteins identified in both (Igo1p, Ngr1p, and Tif4632p) [57]. A listing of PHG kinase-dependent phosphorylation sites in these RNP granule proteins is indicated in Table 1.

**Table 1:** mRNP components differentially phosphorylated in kinase-dead mutants.

Protein	Identified Phosphosite(s)^*a*^	KD/WT (normalized)^b^	Kinase-dead mutant
Dcp2p	S747/ S751/ S771/ S439	4.3/ 1.8/ 0.44/ 2.6	*kss1*/ *fus3*/ *ste20*/ *snf1*
Ded1p	S369	4.9	*ste20*
Dhh1p	S14	0.34	*elm1*
Eap1p	S30/ S282/ T391	0.31/ 0.12/ 0.28	*snf1*
Edc3p	S255	1.8	*fus3*
Hrp1p	S2, S3	2.7	*ste7*
Igo1p	S157	0.03/ 0.08	*snf1*/ *tpk2*
Ngr1p	S524	0.17	*snf1*
Pat1	S255	4.5	*ste20*
Pbp1	S106/ S436	0.32/ 2.9	*elm1*/ *kss1*
Sbp1	T91	3.2/ 2.6/ 3.1/ 5.1/ 8.1	*snf1*/ *ste7*/ *ste11*/ *ste20*/ *tpk2*
Tif4632p	S74/ T355	1.8/ 2.6	*fus3*/ *elm1*
Xrn1p	S1510	2.9	*kss1*
Ygr250cp	S482, T486/ S501/ T653	4.7/ 7.2/ 4.2	*ste20*/ *snf1*/ *ste20*

^a^Residues on distinct phosphopeptides are separated by slashes; residues on a single phosphopeptide are separated by commas. Phosphorylation sites were localized with *p*>0.75.

^b^Ratio of phosphopeptide in kinase-dead mutant relative to wild-type normalized to protein level. All results exhibit a statistical significance of ≤0.05.

As a further step towards identifying a regulatory link between pseudohyphal growth kinase signaling and RNP biology, we constructed a network connectivity map integrating physical interactions between: 1) RNP components and 2) proteins in signaling pathways/cell processes required for filamentation. Using annotations from the Kyoto Encyclopedia of Genes and Genomes (KEGG), we identified a fairly dense map between proteins localized to RNPs and the indicated KEGG pathways related to filamentous growth (Fig 3C). We did not observe maps of similar density between RNP components and proteins in other filamentous growth-related KEGG pathways. The proteins used to generate this network and the database sources of the interactions are listed in S4 Table.

### A set of pseudohyphal growth kinases localizes with RNP particles

**Fig 4:**
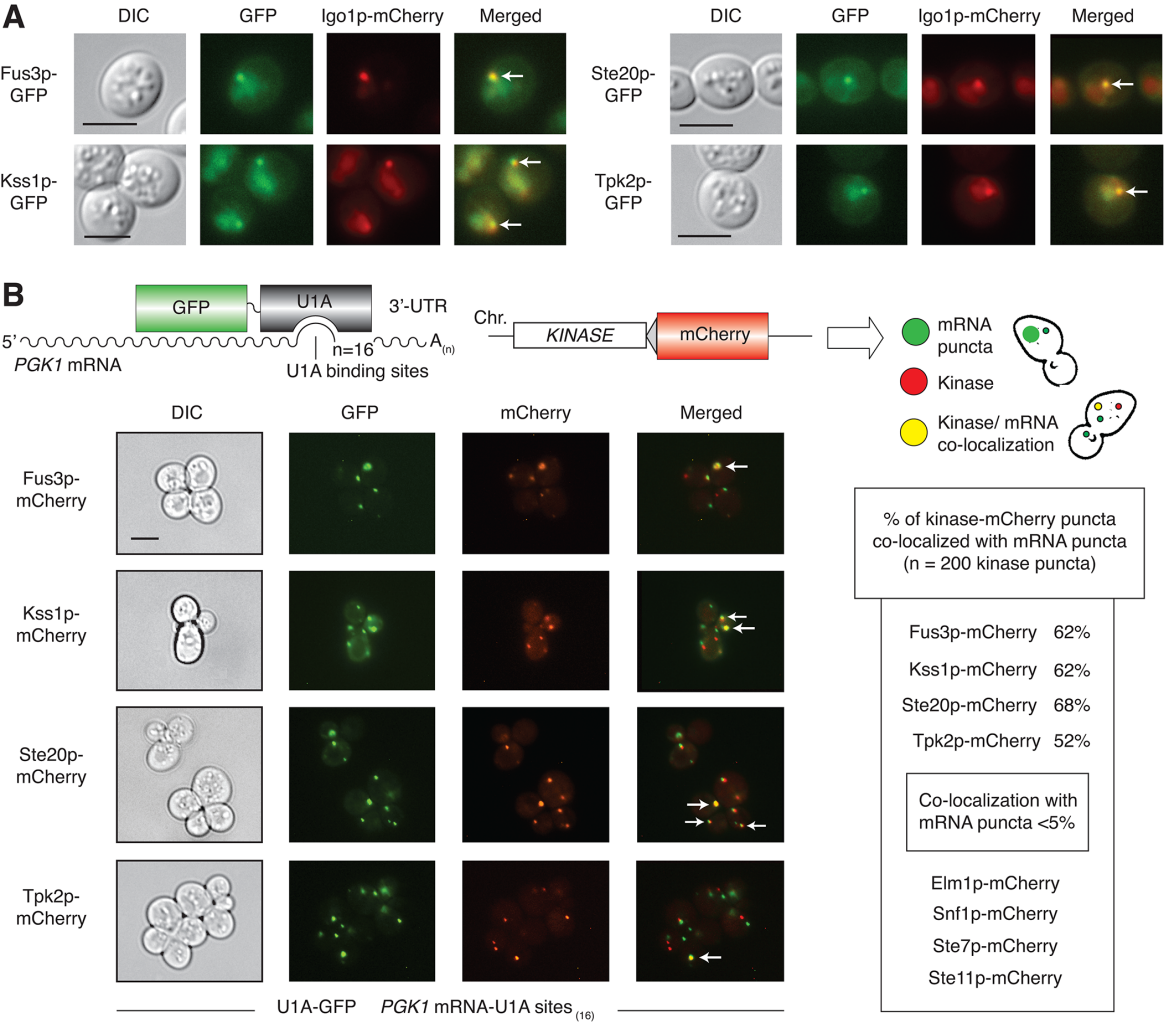
Co-localization of Fus3p, Kss1p, Ste20p, and Tpk2p with mRNPs. A) The RNP component Igo1p was visualized as a carboxy-terminal mCherry fusion generated by integration of a mCherry cassette at the 3’-end of *IGO1*. GFP chimeras were constructed as integrated in-frame fusions to the 3’-ends of the indicated kinase genes. Cells were examined after three days of incubation with shaking in liquid cultures of minimal medium with normal levels of ammonium sulfate. Arrowheads indicate foci with the given kinase and Igo1p. Scale bar, 3 μm. Fluorescent protein fusions to the kinases Elm1p, Snf1p, Ste7p, and Ste11p did not localize significantly as puncta under identical conditions (S3A Fig), and were not tested further for co-localization with Igo1p. B) RNPs were visualized as foci using *PGK1* modified to contain 16 binding sites for U1A-GFP in its 3’-UTR. GFP-tagged RNA was analyzed for co-localization with kinase-mCherry fusions generated by integration of sequence encoding mCherry as an in-frame fusion to the 3’-end of the targeted kinase gene. Kinase localization was observed after 15 minutes of glucose stress in SC–Leu–Ura media lacking glucose. Arrowheads indicate kinase-mCherry puncta co-localized with GFP-tagged RNA foci. Quantification of puncta is provided.

Since the phosphorylation of numerous RNP components is dependent upon pseudohyphal growth kinases, we examined the set of eight kinases selected here for co-localization with RNP particles. For this analysis, we generated carboxy-terminal GFP-fusions to each kinase and carboxy-terminal mCherry fusions to several RNP components (Dcp2p, Edc3p, Igo1p, and Pat1p) as chromosomal alleles in a strain of the filamentous Σ1278b genetic background. As indicated in Fig 4A, Fus3p, Kss1p, Ste20p (in the MAPK pathway) and Tpk2p (PKA) co-localized as GFP chimeras with Igo1p-mCherry. Igo1p is a RNP component of unknown function that has been identified as a Rim15p target required for proper initiation of G_0_ [58]. Rim15p, acting downstream of PKA, phosphorylates Igo1p at S64; this phosphorylation site was also observed in the mass spectrometry studies reported here. Igo1p binds the P-body protein Dhh1p and the stress-granule protein Pbp1p as determined previously by co-immunoprecipitation [58]. We observe Igo1p-mCherry puncta and co-localization with Fus3p, Kss1p, Ste20p, and Tpk2p post-diauxic shift, upon 3 days growth in standard media (Fig 4A).

We further considered co-localization of pseudohyphal growth kinases with RNPs using the RNA visualization strategy of Brodsky and Silver [59]. By this method, RNA can be visualized in puncta by fluorescence microscopy of a U1A-GFP fusion bound to multiple U1A-binding sites introduced into the 3’-untranslated region of a target mRNA. For this study, we used *PGK1* mRNA modified with sixteen U1A-binding sites as a marker of bulk and stable mRNAs (Fig 4B). As described in Sheth and Parker [60], this fluorescence-tagging strategy has been used to visualize RNPs as puncta, and we have co-localized Pbp1-GFP with U1A-mCherry-bound RNA puncta by this approach. Each pseudohyphal growth kinase tested in this study was analyzed for co-localization with *PGK1* RNA puncta, and, consistent with the results presented above, we observed substantial RNA puncta co-localization with mCherry fusions to Fus3p, Kss1p, Ste20p, and Tpk2p (Fig 4B). In contrast, mCherry chimeras with Elm1p, Snf1p, Ste7p, and Ste11p did not co-localize with the engineered *PGK1* RNA. Kinase puncta were evident under a short period of glucose limitation, with RNA co-localization persistent through at least two days. Transferring cells from media limited in glucose to media with normal levels of glucose resulted in a loss of observed puncta, indicating that punctate formation was responsive to glucose limitation.

### RNP components are required for wild type signaling through the pseudohyphal growth PKA and MAPK pathways

**Fig 5:**
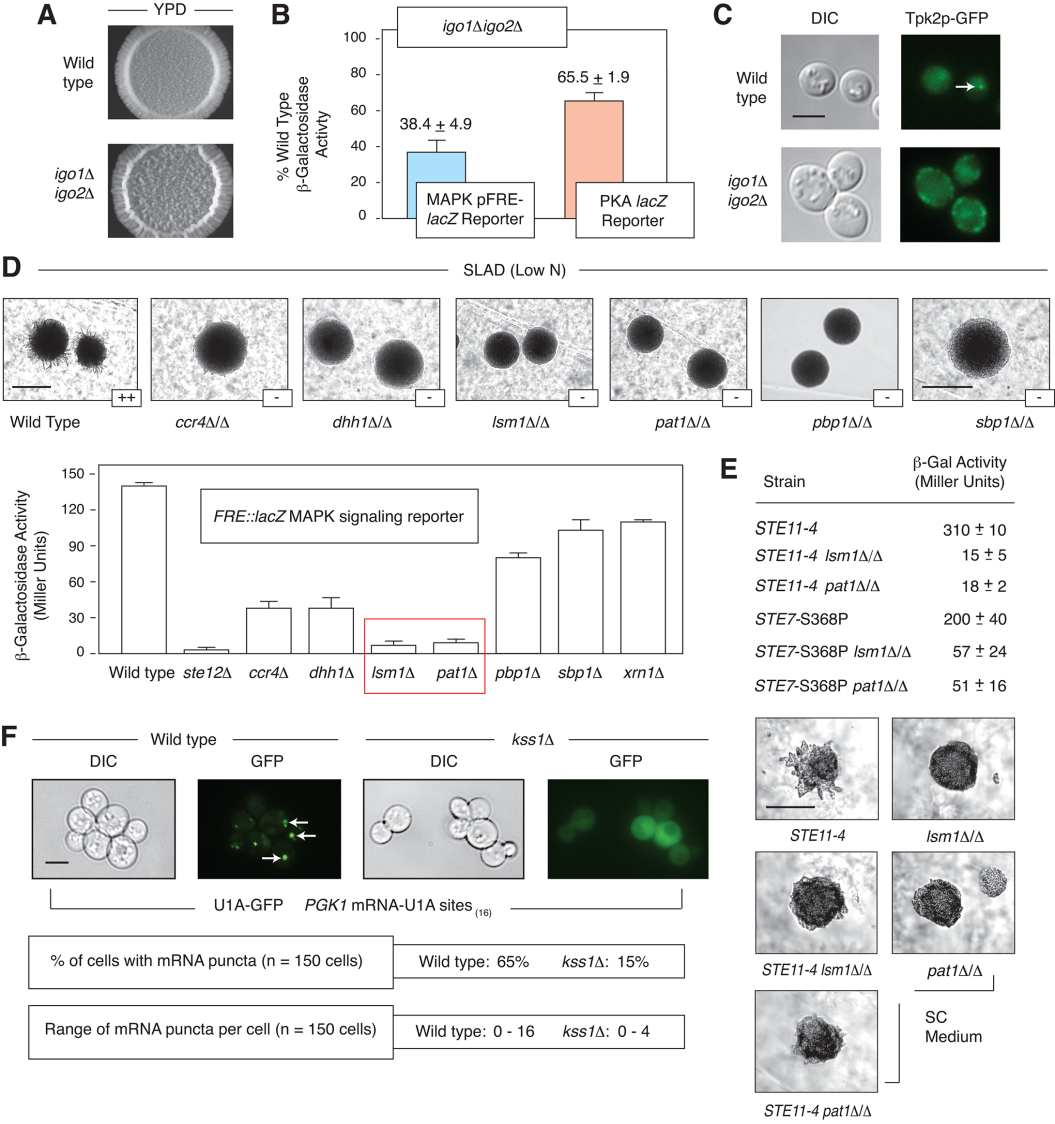
Interrelationship between mRNP components, PKA, and pseudohyphal growth MAPK signaling. A) Morphology of a spotted culture of a haploid strain of the filamentous Σ1278b background deleted for *IGO1* and *IGO2*. Exaggerated wrinkling is evident in the *igo1*Δ*igo2*Δ strain grown on YPD medium. Images of the *igo1*Δ*igo2*0394 strain on low nitrogen SLAD media are presented in S3B Fig. B) FRE- and PKA-regulated (*P*_*flo11-6/7*_) *lacZ* reporters indicate decreased signaling in a haploid *igo1*Δ*igo2*Δ mutant in normal media. Error bars indicate the standard deviation from three biological replicates. C) *IGO1* and *IGO2* are required for wild-type localization of a Tpk2p-GFP fusion in puncta after three days growth in standard SC media. Sequence encoding GFP was fused to the 3’-end of *TPK2* at its native chromosomal locus. The arrowhead identifies a Tpk2p-GFP punctum. Puncta were observed in less than 15% of cells for the *igo1*Δ*igo2*Δ strain (n = 200 cells). Scale bar, 3 μm. D) Surface-spread pseudohyphal filamentation phenotypes are observed in homozygous diploid strains deleted for P-body genes involved in mRNA decay (*CCR4*, *DHH1*, *LSM1*, *PAT1*, *PBP1*, and *SBP1*). Surface spread filamentation was assayed on low nitrogen (SLAD) medium. Scale bar, 2 mm. The degree of filamentation is indicated as “++” (strong surface filamentation) or “-” (decreased surface filamentation). Activity of the pseudohyphal growth Kss1p MAPK signaling pathway is diminished in haploid strains deleted for P-body-localized mRNA decay genes. MAPK pathway activity was assessed using a plasmid-based FRE-*lacZ* reporter. Strains lacking the decapping proteins Lsm1p and Pat1p yielded particularly low levels of *lacZ* signal. Assays were performed on liquid cultures in log phase in standard growth medium. Error bars indicate the standard deviation from three independent trials. E) Epistasis studies of *PAT1* and *LSM1* with the *STE11* and *STE7* genes in the pseudohyphal growth MAPK pathway. Surface filamentation is shown for strains grown on synthetic complete medium. Scale bar, 2 mm. Activity of the pseudohyphal growth Kss1p MAPK pathway as determined using the *FRE*::*lacZ* reporter mirrored the observed colony morphology phenotypes, with the effect slightly lessened in the *STE7*-S368P mutants. The standard deviation in β-galactosidase levels from three independent trials is shown for each strain. F) The pseudohyphal growth MAPK Kss1p is required for wild-type numbers of GFP-tagged RNA puncta as visualized by U1A-GFP binding under conditions of glucose limitation. Arrowheads indicate RNA puncta. Scale bar, 3 μm.

The RNP protein Igo1p is PKA-regulated, and we find that wild-type localization of the PKA catalytic subunit Tpk2p requires *IGO1* and its paralog *IGO2*. The *IGO1* and *IGO2* genes arose from a whole genome duplication event, with 58% identity between these proteins [58], suggesting that these genes are functionally redundant. Upon deleting both genes, complex colony morphology is exaggerated, consistent with perturbed PKA signaling [61] (Fig 5A). *IGO1* and *IGO2* are required for signaling through the Kss1p MAPK pathway and PKA pathway as assessed using *lacZ* reporters containing FRE sites responsive to Kss1p-regulated Ste12p/Tec1p and a segment of the *FLO11* promoter bound by PKA-regulated Flo8p, respectively (Fig 5B). Further, the punctate localization of a Tpk2p-GFP chimera is disrupted in a strain deleted for *IGO1* and *IGO2* (Fig 5C). The subcellular distribution of Kss1p, Fus3p, and Ste20p is unaffected in an *igo1*Δ/*igo2*Δ mutant in the filamentous Σ1278b background (S3C Fig).

Numerous genes encoding RNP components are required for wild-type pseudohyphal growth. We identified strongly diminished pseudohyphal growth in strains of the filamentous Σ1278b background containing homozygous deletions of the mRNA decay/translational repressor genes *CCR4*, *DHH1*, *LSM1*, *PAT1*, *PBP1*, and *SBP1* (Fig 5D). To assess activity of the MAPK pathway in these mutants, we introduced the plasmid-based *FRE-lacZ* Kss1p reporter and assayed for β-galactosidase activity under conditions of nitrogen limitation. As indicated in Fig 5D, *lsm1*Δ/Δ and *pat1*Δ/Δ mutants were strongly decreased in MAPK pathway activity relative to wild type, approaching levels observed in a homozygous diploid strain deleted for *STE12*. *LSM1* and *PAT1* both encode P-body proteins that function in mRNA decapping [60]; Lsm1p functions with a group of six other Lsm family proteins in a complex that associates with the Pat1p decapping enzyme, and, collectively, the proteins mediate mRNA decay through decapping [62]. Epistasis studies indicate that overactive alleles of *STE11* and *STE7* [63] are suppressed by deletion of *LSM1* and *PAT1*, both morphologically and by *FRE*-*lacZ* reporter assay (Fig 5E). A hyperactive *KSS1* allele did not yield an exaggerated pseudohyphal growth phenotype. These results indicate that the core mRNA decapping proteins Lsm1p and Pat1p are required for wild-type pseudohyphal growth MAPK signaling, and that the genes act at or below the level of the MAPKK *STE7*.

### The pseudohyphal growth MAPK Kss1p is required to achieve wild type numbers of RNA puncta

In addition to the requirement for RNP components in wild-type Kss1p signaling, we find that *KSS1* is required to achieve wild-type levels of mRNA puncta. A strain of the filamentous Σ1278b background deleted for *KSS1* yields a significantly decreased amount of U1A-GFP-tagged *PGK1* mRNA localized in puncta under conditions of glucose limitation (Fig 5F). This phenotype was consistent in the *kss1*Δ strain from a brief 15-minute incubation under conditions of glucose limitation up to a period of at least eight hours. The percentage of cells exhibiting mRNA puncta decreased from 65% in wild-type cells to 15% in *kss1*Δ mutants, and, correspondingly, the maximum number of puncta observed in these cells decreased from maximally 16 in wild-type to no greater than four in the *kss1*Δ strain. Visible puncta were lost upon the introduction of media with normal levels of glucose. Profiling studies indicate that bulk RNA association with polyribosomes is not substantially altered in a strain of the Σ1278b background deleted for *KSS1* (S4 Fig), although the translational processing of specific transcripts may still be perturbed in a *kss1*Δ strain.

### The RNP-localized Dhh1p helicase is required for hyphal development in *Candida albicans*

**Fig 6:**
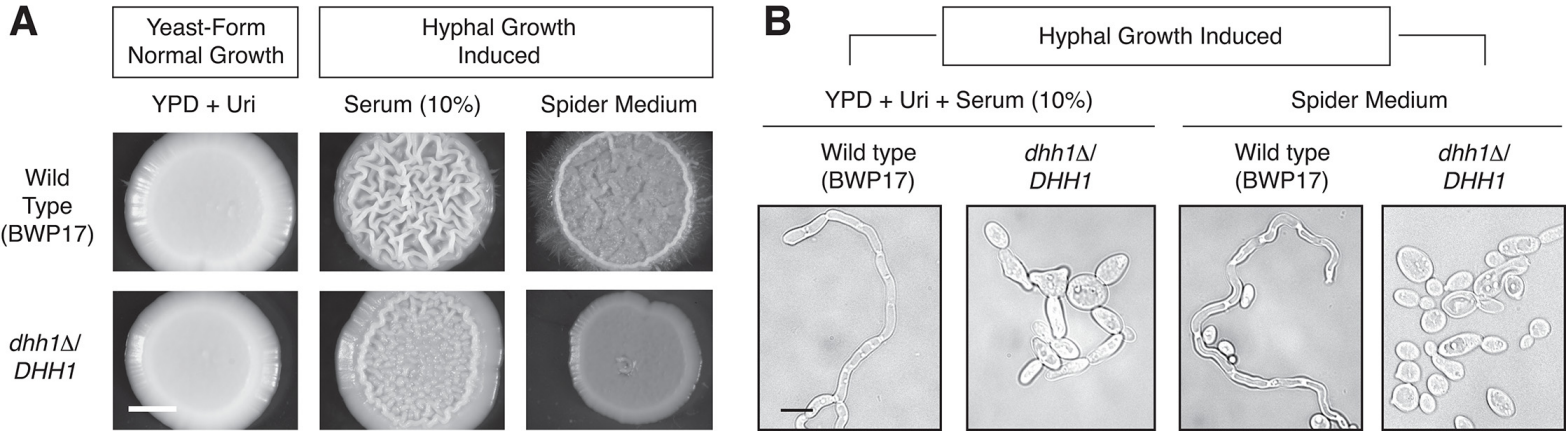
The P-body-localized mRNA decay gene *DHH1* is required for wild-type hyphal development in the opportunistic human fungal pathogen *Candida albicans*. A) The colony morphology of a heterozygous *dhh1*Δ/*DHH1* mutant exhibits diminished surface wrinkling and decreased peripheral filamentation relative to wild type under conditions inducing hyphal growth (the presence of 10% serum or Spider Medium with 1% mannitol at 37°C). A mutant phenotype was not observed in the *dhh1*Δ/*DHH1* mutant under conditions promoting yeast-form growth (YPD supplemented with uridine at 30°C). Scale bar, 1 mm. B) Cells of the *dhh1*Δ/*DHH1* mutant are less elongated than wild-type under conditions inducing hyphal growth. Scale bar, 3 μm.

To consider the likelihood of a conserved functional interrelationship between RNP components and filamentous growth, we assessed the contributions of the P-body protein Dhh1p towards hyphal development in the pathogenic fungus *Candida albicans*. We selected *DHH1* for study because it is a core component of P-bodies, and its localization has been confirmed in *C*. *albicans*. Further, in *S*. *cerevisiae*, a loss-of-function mutation in *DHH1* results in decreased pseudohyphal growth (Fig 5D), although its hyphal growth phenotype in *C*. *albicans* has not been clearly identified. The *C*. *albicans* ortholog of *DHH1* is presumed to function similarly to *S*. *cerevisiae DHH1*, and the genes exhibit 91% sequence similarity at the encoded amino acid level. For this analysis, we generated a heterozygous deletion of *DHH1* in *C*. *albicans* by standard gene replacement and assayed for altered colony and cell morphology under conditions inducing hyphal development. As indicated in Fig 6A, the *dhh1*Δ/*DHH1* strain exhibits reduced surface wrinkling and peripheral hyphae relative to an isogenic wild-type strain. Deletion of *DHH1* resulted in cells that were less elongated than wild type, and, correspondingly, hyphae were decreased in number under these conditions (Fig 6B).

## Discussion

The phosphoproteomic analysis presented here is the first such study of kinase signaling networks in pseudohyphal growth, effectively complementing previous phosphoproteomic analyses of yeast kinases in a non-filamentous strain under standard growth conditions. Compared to data in the major phosphorylation databases (Experimental Procedures), this analysis identifies a large set of previously unreported phosphorylation sites. These sites encompass residues that are phosphorylated strictly in the Σ1278b proteome in response to the conditions employed here as well as phosphorylation sites that were not sampled in previous analyses. This suggests that the quantitative phosphoproteomic data collected here and elsewhere are not saturating. Consequently, there is benefit in continued analysis of the yeast phosphoproteome towards understanding kinase networks more fully.

Our mass spectrometry data identify 73 proteins that: 1) were differentially phosphorylated in a kinase-dead strain, and 2) result in a pseudohyphal growth phenotype upon deletion. Ryan *et al*. [39] reported 497 genes that result in diploid pseudohyphal growth defects in *S*. *cerevisiae* under conditions of low nitrogen; thus, the kinase signaling network identified here encompasses a significant fraction of this gene set. The network contains both direct and indirect kinase targets. In a landmark study, Ptacek *et al*. [64] used a proteome microarray to identify in vitro substrates of most yeast kinases. We integrated the results from this microarray study for the kinases tested here with our own data, identifying in vitro substrates that also exhibited differential phosphorylation by mass spectrometry. The results are indicated in S5 Table.

Further interpretation of the kinase-dependent phosphorylation data yields two observations. First, many of the identified phosphorylation sites were dependent on the presence of more than one pseudohyphal growth kinase. This observation holds true for kinases that are thought to function in distinct pathways. Of 311 unique proteins identified as undergoing differential phosphorylation in a strain carrying a kinase-dead mutation in the MAPK Kss1p, 188 of these proteins were also differentially phosphorylated in a strain carrying a kinase-dead allele of *ELM1*. We report 814 unique proteins differentially phosphorylated in *tpk2-K99R*, and 249 of these proteins were also differentially phosphorylated in a strain with a kinase-dead allele of *KSS1*. Collectively, this suggests that the respective kinases and pathways regulate partially overlapping signaling networks. Overlapping signaling networks further suggest a degree of functional redundancy, although mutant phenotypes are evident upon deletion of each individual kinase analyzed here. Second, the identification of new and functionally important phosphorylated residues in pseudohyphal growth proteins such as Flo8p underscores the utility in extending quantitative phosphoproteomic studies to the analysis of non-standard growth conditions and strains, as *FLO8* is an incompletely translated pseudogene in standard S288C laboratory strains of *S*. *cerevisiae*.

The functional interrelationship identified in this study between RNP components and pseudohyphal growth is supported by several lines of evidence obtained in the non-filamentous S288C genetic background. As reported in Yoon *et al*. [65], Ste20p phosphorylates Dcp2p at Ser 137, and this phosphorylation is required for Dcp2p localization in P-bodies. Shah *et al*. [66] found that overexpression of PKA isoforms inhibits P-body formation and that the Tpk1p and Tpk2p subunits of PKA are capable of phosphorylating the P-body protein Pat1p in vitro. Also in an S288C background under conditions of glucose depletion, Xrn1p undergoes Snf1p-dependent phosphorylation along with a subset of additional mRNA processing proteins [48]. In S288C, Fus3p co-localizes with P-bodies and stress granules as yeast cells enter stationary phase [67]. It is noteworthy that Kss1p is non-functional in the S288C strain [26] and presumably would not have been identified as a regulator of RNPs in previous studies undertaken in that genetic background.

In the filamentous Σ1278b strain, the kinases Kss1p, Fus3p, Tpk2p, and Ste20p co-localize with *PGK1* mRNA foci as well as with the P-body and stress-granule protein Igo1p. As presented in Buchan *et al*. [68], mRNAs are thought to traffic between P-bodies, stress granules, and other RNPs, with the composition of each particle being dynamic in response to the specific cell stress and duration of the stress. Since we do not at present understand the full composition of these particles in a filamentous strain under the observed growth conditions, we use the term RNP here to indicate co-localization with RNA/protein foci and specifically identify co-localization with Igo1p.

It is interesting that the upstream PAK Ste20p and the MAPK Kss1p co-localize with Igo1p and GFP-tagged mRNA, but neither the MAPKKK Ste11p nor the MAPKK Ste7p share this localization pattern. Three points are relevant in considering the localization of these pathway components. First, prior literature supports the observation that MAPK pathway components may not be uniformly localized. For example, in the mating pathway, Ste7p and Fus3p have been identified at the bud tip, although Ste11p has not been similarly localized. Fus3p exists in several complexes affecting its localization [69]. Kss1p has been reported previously to localize to the nucleus [70], although neither Ste11p nor Ste7p are found predominantly in the nucleus. Second, this work and other studies [64, 71] indicate that the respective MAPK pathway components do not exhibit strictly overlapping targets. The fact that these kinases recognize nonoverlapping sets of targets suggests that differential localization of subpopulations of the kinases is possible. Third, Kss1p and Fus3p as well as the pseudohyphal growth upstream PAK Ste20p are not exclusively identified in mRNPs, and the likelihood exists that a subset of these respective protein populations may indeed be colocalized.

RNPs are induced in response to numerous cell stresses, including glucose limitation, hyperosomotic stress, and high cell density [72–74]; however, we find that nitrogen stress, a classic inducer of pseudohyphal growth, is not a strong inducer of RNPs in the absence of additional cell stresses. The mechanisms of these inductions are unclear; consequently, it is also unclear as to why nitrogen limitation alone is insufficient to strongly induce this response. In this analysis, 1-butanol, rather than low-nitrogen media, was used as an inducer of filamentation because of its ability to yield a strong filamentous response in liquid cultures. Further, the cells necessitated growth to 10 doublings under conditions inducing pseudohyphal growth to achieve efficient labeling, which resulted in a mild degree of glucose stress. The presence of short-chain alcohols coupled with glucose limitation provides strong induction of filamentation in liquid. Notably, we observe RNP foci under these conditions for a growth period of at least 30 hours, representing a time point matching the endpoint of growth for quantitative phosphoproteomic analysis. It should be noted that stress granules form post entry into stationary phase [66], and consequently, this analysis may be less effective in identifying post-translational modifications affecting stress granule components. In sum, however, the conditions present at the point of mass spectrometric analysis allow for the presence of RNPs, consistent with the identification of differentially phosphorylated RNP components in this study.

Interestingly, the requirement for *DHH1*, encoding a helicase involved in mRNA decapping, is conserved between *S*. *cerevisiae* and the related pathogenic fungus *C*. *albicans*. Signaling pathways in *S*. *cerevisiae* serve as effective models of related pathways in *C*. *albicans* [75], raising the possibility that RNPs and hyphal development are functionally linked in *C*. *albicans* as well. In support of this notion, P-bodies have been observed to form during hyphal development in *C*. *albicans*, and Dhh1p does co-localize to P-bodies in *Candida* [76]. Further, a strain of *C*. *albicans* deleted for *EDC3* exhibits a defect in filamentation [76]. The results here are consistent with regulatory feedback between RNP components and hyphal development in this opportunistic human pathogen.

In total, the data support an interrelationship between pseudohyphal growth kinase signaling and RNP biology. Deletion analyses and epistasis studies indicate that RNA processing proteins are required for wild-type Kss1p MAPK signaling, likely to regulate the translational state of particular transcripts important for pseudohyphal growth. In turn, the identified pseudohyphal growth kinases localize to RNPs, and the Kss1p pathway is required for wild-type RNP numbers in the filamentous *S*. *cerevisiae* strain. Thus, Kss1p MAPK signaling and RNP signaling feed back reciprocally. Similarly, PKA regulates Igo1/2p function through Rim15p, and Igo1/2p is in turn required for wild-type PKA localization. The data here collectively provide an important step towards identifying the mechanisms through which this reciprocal signaling is mediated.

## Materials and Methods

### Strains, plasmids, and media

Strains used in this study are listed in S6 Table. *S*. *cerevisiae* strains were derived from the filamentous Σ1278b genetic background. Haploid strains were derived from Y825 and HLY337 [1, 77]. Standard protocols and techniques were used for the propagation of budding yeast as described [78]. DNA was introduced by methods of yeast transformation incorporating lithium acetate treatment and heat shock [79]. Plasmids used in this study are listed in S7 Table.

*S*. *cerevisiae* strains were cultured on YPD (1% yeast extract, 2% peptone, 2% glucose) or Synthetic Complete (SC) (0.67% yeast nitrogen base (YNB) without amino acids, 2% glucose, and 0.2% of the appropriate amino acid drop-out mix). Nitrogen deprivation and filamentous phenotypes were assayed using Synthetic Low Ammonium Dextrose (SLAD) medium (0.17% YNB without amino acids, 2% glucose, 50 μM ammonium sulfate and supplemented with appropriate amino acids) or by supplementing growth medium with 1% 1-butanol [9]. Glucose limitation was achieved using media lacking glucose as a carbon source according to standard protocols.

### Generation of gene deletions and integrated site-directed mutants

Gene deletions and tags for chromosomal integration were generated through one-step PCR- mediated transformation and subsequent PCR-based verification [80, 81]. N-terminal and C-terminal GFP tagging was performed using plasmid-based modules from Longtine *et al*. [82]. Carboxy-terminal mCherry tagging was performed by PCR-based amplification of the mCherry-*kanMX* or *Hyg* ^R^ cassette of pBS34 or pBS35 (Yeast Resource Center, Univ. of Washington). Integrated point mutations in *RAS2* (*Y165F*, *T166A*) and *FLO8* (*S587A*,*S589A*, and *S590A*) were generated by the *URA* “flip-out” method. In brief, the *URA3* cassette of pRS406 was amplified by PCR and used to disrupt local sequence at the intended site of mutation. A second transformation was then performed to replace the *URA3* marker with DNA sequence encoding the desired mutated allele [5].

### Experimental design and cell labeling for phosphoproteomic analysis

*ARG4* and *LYS1* were deleted in the haploid Y825 filamentous strain. Subsequently, protein kinase genes were individually deleted in the filamentous *arg4*Δ *lys1*Δ auxotrophic Y825 background. These arginine/lysine auxotrophic kinase null strains were then transformed with *LEU2*-containing yeast shuttle vectors carrying the respective kinase-dead alleles. SILAC was used to differentially label proteins synthesized by kinase-dead allele strains and wild-type (*arg4*Δ *lys1*Δ Y825). SILAC-based mass spectrometry experiments were multiplexed; each mass spectrometry experiment was conducted in triplex, with the light (natural) versions of L-arginine and L- lysine used to label the wild-type strain and medium (Lys-4/Arg-6) or heavy (Lys-8/Arg-10) L-arginine and L-lysine labeling two kinase-dead allele strains.

In each triplex experiment, three strains were cultured in parallel during filamentous growth-inducing conditions. Wild-type and two kinase-dead allele strains were cultured overnight in synthetic complete media containing arginine or lysine residues with light, medium, or heavy isotopes overnight at 30°C, to obtain actively growing log-phase cultures. Each culture was then diluted to a low starting optical density (OD_600_ of approximately 0.1) in SILAC media. To induce filamentous growth in these haploid strains, 1% (vol/vol) 1-butanol was added to each culture. These diluted cultures were subsequently incubated at 30°C for approximately 10 doublings (approximately 26 hours). This prolonged labeling and culturing step was found to be necessary to ensure effective metabolic labeling of proteins, as well as to obtain a minimum abundance of labeled protein from each strain. Protein extractions and mass spectrometry were performed as described previously [42, 83].

### Mass spectrometry data analysis and network construction

We processed mass spectrometry data using maxQuant [84] and collated the list of phospho (STY) peptides. The data were filtered at 5% FDR; additionally, we excluded peptides exhibiting low Mascot scores (<3), high charge states (≥5), and long peptide lengths (>40). A normalized heavy:light or medium:light ratio with a significance score (Sig A) ≤ 0.05 was considered statistically significant. Predicted phosphorylation sites were screened for known kinase motifs and the results from this analysis are included in the “Motifs” column in S2 Table.

To identify potentially novel phosphorylation sites, Exonerate was used to align peptides extracted from major phosphorylation databases (GPM DB, PhosphoPep, PHOSIDA, and Phospho.ELM) onto yeast protein sequence data from the Saccharomyces Genome Database. Phosphosites were marked on the protein sequences, yielding a compendium of phosphorylation sites. Inherent ambiguities in the localization of phosphorylation sites were annotated as such in the compendium. Phosphorylation sites identified in our data were subsequently mapped onto the annotated phosphoproteome.

Networks were constructed using background sets of protein interactions encompassing kinase-dependent differentially phosphorylated proteins in the mass spectrometry data generated here as well as interactions identified in the iRefIndex database. KEGG signaling pathways relevant for pseudohyphal growth were downloaded and parsed using in-house scripts. The resulting network was expanded by including previously identified core components of stress granules and P-bodies [57, 85]. The network was visualized using Cytoscape. The interactions used to construct this network and the database source of each interaction are provided in S4 Table.

### Visualization of mRNA and RNPs by fluorescence microscopy

RNA localization in RNP foci was visualized as described [57]. Live *S*. *cerevisiae* cells with these plasmids and/or fluorescent protein fusions to known mRNP components were imaged using an upright Nikon Eclipse 80i microscope with CoolSnap ES2 CCD (Photometrics). Images were acquired using the MetaMorph software package (Molecular Devices).

### *C*. *albicans* strains and analysis of filamentous development

*Candida albicans* strains used in this study were derived from the CAI4 genetic background (*ura3*Δ::*imm434*/*ura3*Δ::*imm434*). The *DHH1*/*dhh1*Δ heterozygote was generated independently by two approaches: 1) by replacement of one endogenous *DHH1* allele with a *URA3* cassette, and 2) by allele replacement using a *HIS1* cassette. A transformant generated by each method was tested for filamentous development, and identical results were observed (Fig 6 and S5 Fig). To induce hyphal formation, strains were inoculated onto standard YEPD plates (2% glucose, 2% peptone, 1% yeast extract) supplemented with 80 mg/L uridine and 1% or 10% fetal calf serum (FCS) as indicated. Hyphal formation was also induced by growth on carbon-limiting Spider medium (10g nutrient broth, 10g mannitol, 2g K_2_HPO_4_ per liter media) [86].

## Supporting Information

S1 Fig Kinase regulation of yeast pseudohyphal growth. A) Kinase signaling pathways that regulate yeast pseudohyphal growth are indicated. Major kinase pathways are boxed. Arrows indicate positive regulation of protein activity, and negative regulation is indicated as interrupted lines. Kinases analyzed in this study are highlighted in red. B) Surface-spread pseudohyphal growth phenotypes are shown for strains carrying kinase-dead alleles of the indicated genes. The degree of surface-spread growth is indicated in the inset boxes, with “++” representing exaggerated filamentous growth and “-” indicating decreased surface-spread filamentation. Strains were grown on low-nitrogen medium (SLAD). Scale bar, 2 mm. C) Colony morphology of yeast strains carrying wild-type and kinase-dead alleles of *FUS3*. The exaggerated morphology of the *fus3* mutant is indicated in the inset box (++) relative to wild type. D) Yeast invasive growth is diminished relative to wild type in a strain with a kinase-dead allele of *KSS1*. Images pre- and post-washing of surface cells are shown.
(TIF)

S2 Fig Phenotypic analysis of site-directed mutants with non-phosphorylatable substitutions at newly identified phosphorylation sites in Ras2p and Flo8p. A) The *ras2*-Y165F T166A mutant exhibits diminished invasive growth (-) on YPD medium relative to wild-type (+). Scale bar, 1 mm. GFP fusions to the amino terminus of wild-type Ras2p and the Ras2p-Y165F T166A mutant localize to the plasma membrane, indicating that the mutant protein is expressed and does localize properly. B) The *flo8*-S3A mutant undergoes decreased invasive growth relative to wild type. A GFP fusion to the carboxy terminus of a Flo8p mutant with Ala substitutions at S587, S589, S590, and S593 (Flo8p-S4A) yields similar levels of fluorescence and nuclear localization patterns to wild-type Flo8p-GFP. The nucleus was visualized in these cells using a Mad1p-NLS-tDimer chimera. Merged images are shown to the right. Scale bar, 3 μm.
(TIF)

S3 Fig PHG kinase localization and *IGO1/2* deletion phenotypes. A) The Elm1p-mCherry, Snf1p-mCherry, Ste7p-GFP, and Ste11p-GFP chimeras do not exhibit significant numbers of puncta after 3 days growth. All chimeras were constructed as integrated in-frame fusions to the 3’-end of each indicated gene. Differential interference contrast (DIC) and fluorescent micrograph images are presented. Scale bar, 3 μm. B) Images of spotted cultures (scale bar, 1 mm) and liquid cultures (scale bar, 3 μm) of a haploid strain deleted for *IGO1* and *IGO2* in low nitrogen SLAD media. A wild type haploid strain is shown for comparison. No changes in pseudohyphal filamentation or cell morphology are evident in the *igo1/2*Δ strain. C) The subcellular localization of the MAPKs Fus3-GFP and Kss1-GFP, as well as the distribution of the upstream PAK Ste20p-GFP are unaffected by deletion of *IGO1* and *IGO2*. Arrows indicate puncta for each kinase. Scale bar, 3 μm.
(TIF)

S4 Fig Polysome fractionation of lysate from yeast strains. A) Analysis of a yeast strain with *RPL38-GFP*. Yeast cells were grown to log phase prior to the addition of cycloheximide at a final concentration of 0.1 mg/ml. The harvested cell pellets were resuspended in a slurry of 20 mM HEPES, 1.2% PVP40, 0.1 mg/ml cycloheximide, and Roche EDTA-free protease inhibitor cocktail prior to freezing in liquid nitrogen. Cell extracts were prepared from the frozen cells using a planetary ball mill under cryogenic conditions. The extracts were dissolved in polysome extraction buffer (20 mM HEPES, 140 mM KCl, 5 mM MgCl2, 0.1 mg/ml cycloheximide, 0.5 mM DTT, and protease inhibitor cocktail). The resulting lysates were centrifuged, and clarified lysates containing equivalent amounts of total RNA from treated cells were layered onto 12-ml continuous linear 7–50% (w/v) sucrose gradients in polysome buffer. Velocity sedimentation was performed by centrifuging the gradients at 35,000 rpm for 4 hours at 4 degrees C in a SW41 rotor. 500 μl fractions were manually collected from the top of the gradient to the bottom. Absorbance at 254 nm was measured for each fraction, and the fractions were analyzed for the presence of Rpl38p-GFP by Western blotting. Proteins were detected using Millipore Luminata Crescendo Western HRP Substrate and a BioRad ChemiDoc XRS imaging system with Image Lab software. In the polysome trace, peaks representing association of the large ribosomal subunit and monosome with mRNA are labeled 60S and 80S respectively. The heavier fractions containing polyribosomes are also identified. The polysome trace is from a single experiment representative of two biological replicates. Western blots of both biological replicates are located under the polysome trace. B) Analysis of the *kss1*Δ *RPL38-GFP* strain was carried out as described above.
(TIF)

S5 Fig Independent construction of a heterozygous *dhh1*Δ*/DHH1* mutant using a *HIS1* cassette results in a consistent phenotype indicating decreased central wrinkling of a spotted culture. Images were obtained on indicated media after two days growth. Scale bar, 1 mm.
(TIF)

S1 Table Listing of pseudohyphal growth phenotypes of kinase-dead mutants studied in this work. (DOCX)

S2 Table Listing of proteins differentially phosphorylated in the kinase-dead mutants. The “Kinase” column indicates the kinase-dead allele in which the differentially phosphorylated protein was identified. The PEP score, Mascot score, and PTM score for each protein record is indicated. The normalized ratio of phosphorylated peptide in kinase-dead mutant versus wild type is provided, along with the significance of the ratio (SigA). Data from the constructed compendium of phosphorylation sites has been integrated as the “Known Site” column; blank indicates that we could not identify the phosphorylation site/peptide in the phosphorylation databases. IDs from the phosphorylation databases are provided in the “locEvi” and “pepEvi” columns when available. PepEvi provides evidence of a peptide match, while “locEvi” indicates the localization of phosphorylation to the indicated residue(s). Predicted phosphorylation sites matching a kinase family motif are indicated in the “Motifs” column; the “Best Motif” column indicates the motif that matches the peptide sequence most strongly.
(XLSX)

S3 Table Listing of Gene Ontology terms enriched in the set of proteins hyper-phosphorylated in one of the kinase-dead mutants tested here. Indicated terms are enriched to a *p*-value of less than 0.001.
(XLSX)

S4 Table Listing of proteins and respective database sources used to construct the signaling network maps in Fig 3. The relevant KEGG pathways for MAPK signaling in yeast (sce04011), cell cycle (sce04111), and meiosis (sce04113) are listed. Each constituent protein is shown with its systematic and common name; KEGG Ortholog group annotations and Enzyme Commission numbers are also listed in brackets. RNP components used to construct the network maps are also listed, with systematic name and common names indicated. The database source of each interaction in Fig 3C is listed as a separate sheet in this file.
(XLSX)

S5 Table Listing of in vitro substrates identified by proteome microarray for the kinases tested here that also exhibited differential phosphorylation in our study in the respective kinase-dead strain. Each unique peptide identified in our analysis for each of these proteins is reported.
(XLSX)

S6 Table Listing of strains used in this study. (DOCX)

S7 Table Listing of plasmids used in this study. (DOCX)
