# Supplementary figures and images for: Large-Scale Analysis of Kinase Signaling in Yeast Pseudohyphal Development Identifies Regulation of Ribonucleoprotein Granules
Source: PLoS Genet. 2015 Oct 8;11(10):e1005564. doi: 10.1371/journal.pgen.1005564 (PMC4598065; doi:10.1371/journal.pgen.1005564)

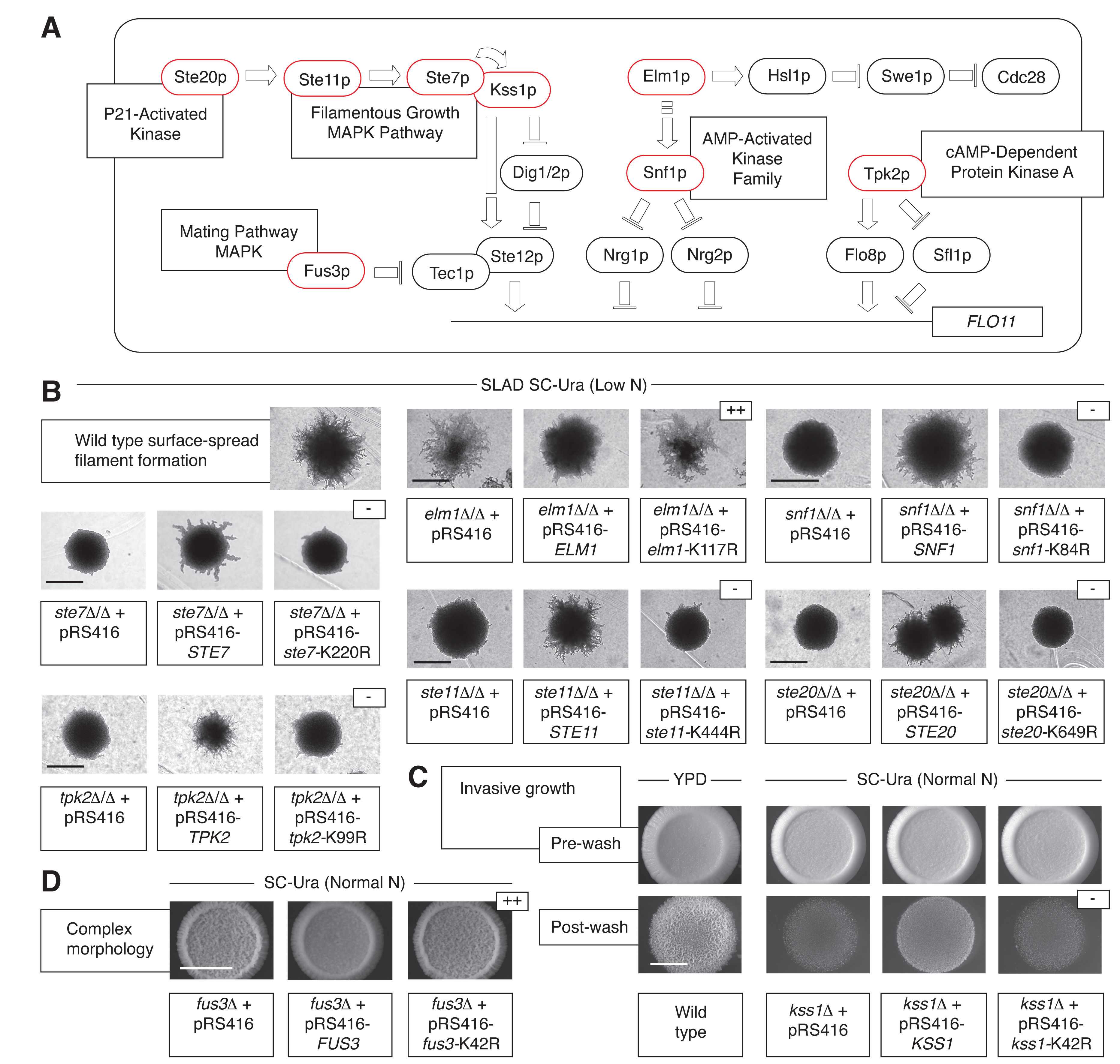

Supplement: S1 Fig — A) Kinase signaling pathways that regulate yeast pseudohyphal growth are indicated. Major kinase pathways are boxed. Arrows indicate positive regulation of protein activity, and negative regulation is indicated as interrupted lines. Kinases analyzed in this study are highlighted in red. B) Surface-spread pseudohyphal growth phenotypes are shown for strains carrying kinase-dead alleles of the indicated genes. The degree of surface-spread growth is indicated in the inset boxes, with “++” representing exaggerated filamentous growth and “-” indicating decreased surface-spread filamentation. Strains were grown on low-nitrogen medium (SLAD). Scale bar, 2 mm. C) Colony morphology of yeast strains carrying wild-type and kinase-dead alleles of FUS3. The exaggerated morphology of the fus3 mutant is indicated in the inset box (++) relative to wild type. D) Yeast invasive growth is diminished relative to wild type in a strain with a kinase-dead allele of KSS1. Images pre- and post-washing of surface cells are shown. (TIF) [file pgen.1005564.s001.tif]

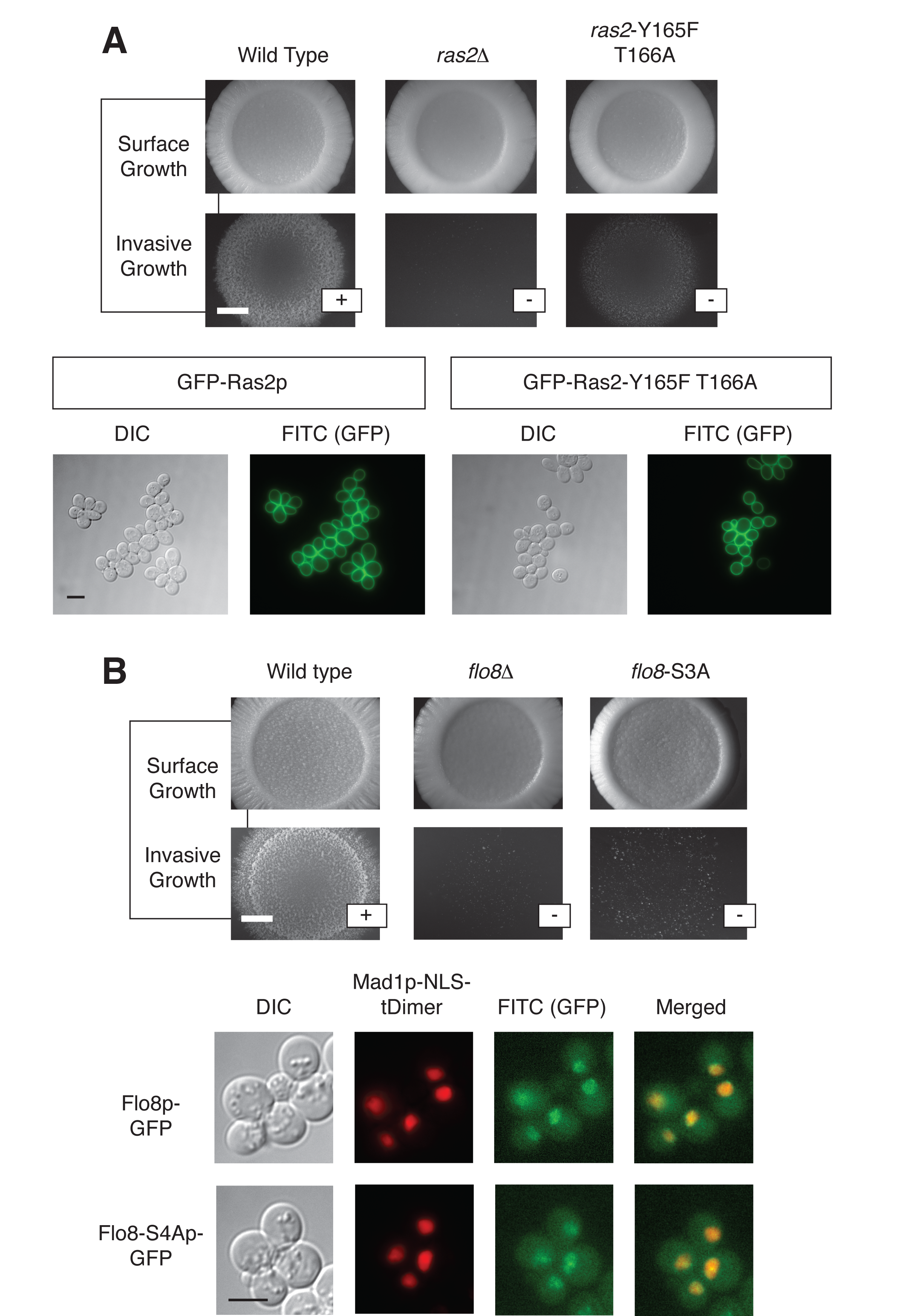

Supplement: S2 Fig — A) The ras2-Y165F T166A mutant exhibits diminished invasive growth (-) on YPD medium relative to wild-type (+). Scale bar, 1 mm. GFP fusions to the amino terminus of wild-type Ras2p and the Ras2p-Y165F T166A mutant localize to the plasma membrane, indicating that the mutant protein is expressed and does localize properly. B) The flo8-S3A mutant undergoes decreased invasive growth relative to wild type. A GFP fusion to the carboxy terminus of a Flo8p mutant with Ala substitutions at S587, S589, S590, and S593 (Flo8p-S4A) yields similar levels of fluorescence and nuclear localization patterns to wild-type Flo8p-GFP. The nucleus was visualized in these cells using a Mad1p-NLS-tDimer chimera. Merged images are shown to the right. Scale bar, 3 μm. (TIF) [file pgen.1005564.s002.tif]

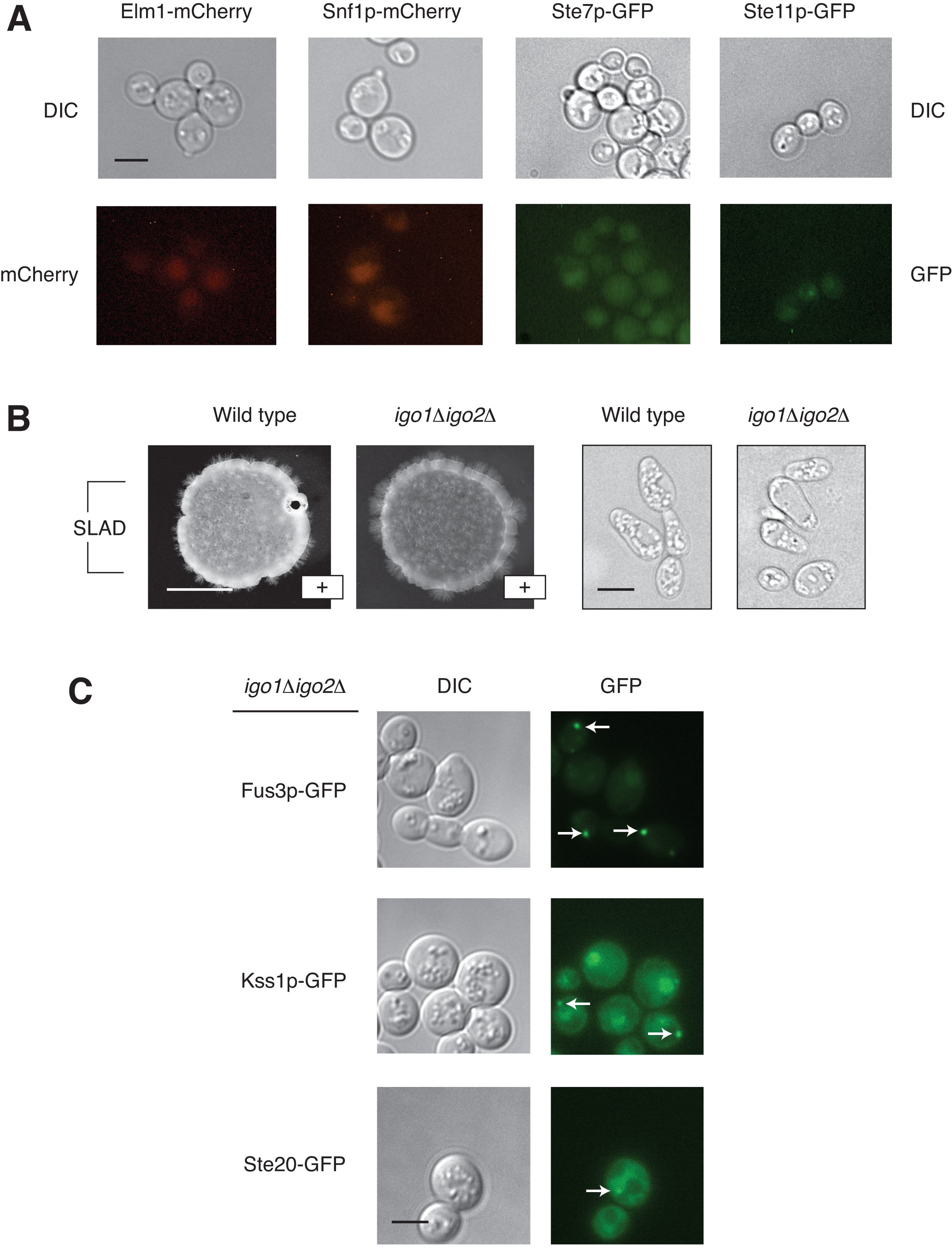

Supplement: S3 Fig — A) The Elm1p-mCherry, Snf1p-mCherry, Ste7p-GFP, and Ste11p-GFP chimeras do not exhibit significant numbers of puncta after 3 days growth. All chimeras were constructed as integrated in-frame fusions to the 3’-end of each indicated gene. Differential interference contrast (DIC) and fluorescent micrograph images are presented. Scale bar, 3 μm. B) Images of spotted cultures (scale bar, 1 mm) and liquid cultures (scale bar, 3 μm) of a haploid strain deleted for IGO1 and IGO2 in low nitrogen SLAD media. A wild type haploid strain is shown for comparison. No changes in pseudohyphal filamentation or cell morphology are evident in the igo1/2Δ strain. C) The subcellular localization of the MAPKs Fus3-GFP and Kss1-GFP, as well as the distribution of the upstream PAK Ste20p-GFP are unaffected by deletion of IGO1 and IGO2. Arrows indicate puncta for each kinase. Scale bar, 3 μm. (TIF) [file pgen.1005564.s003.tif]

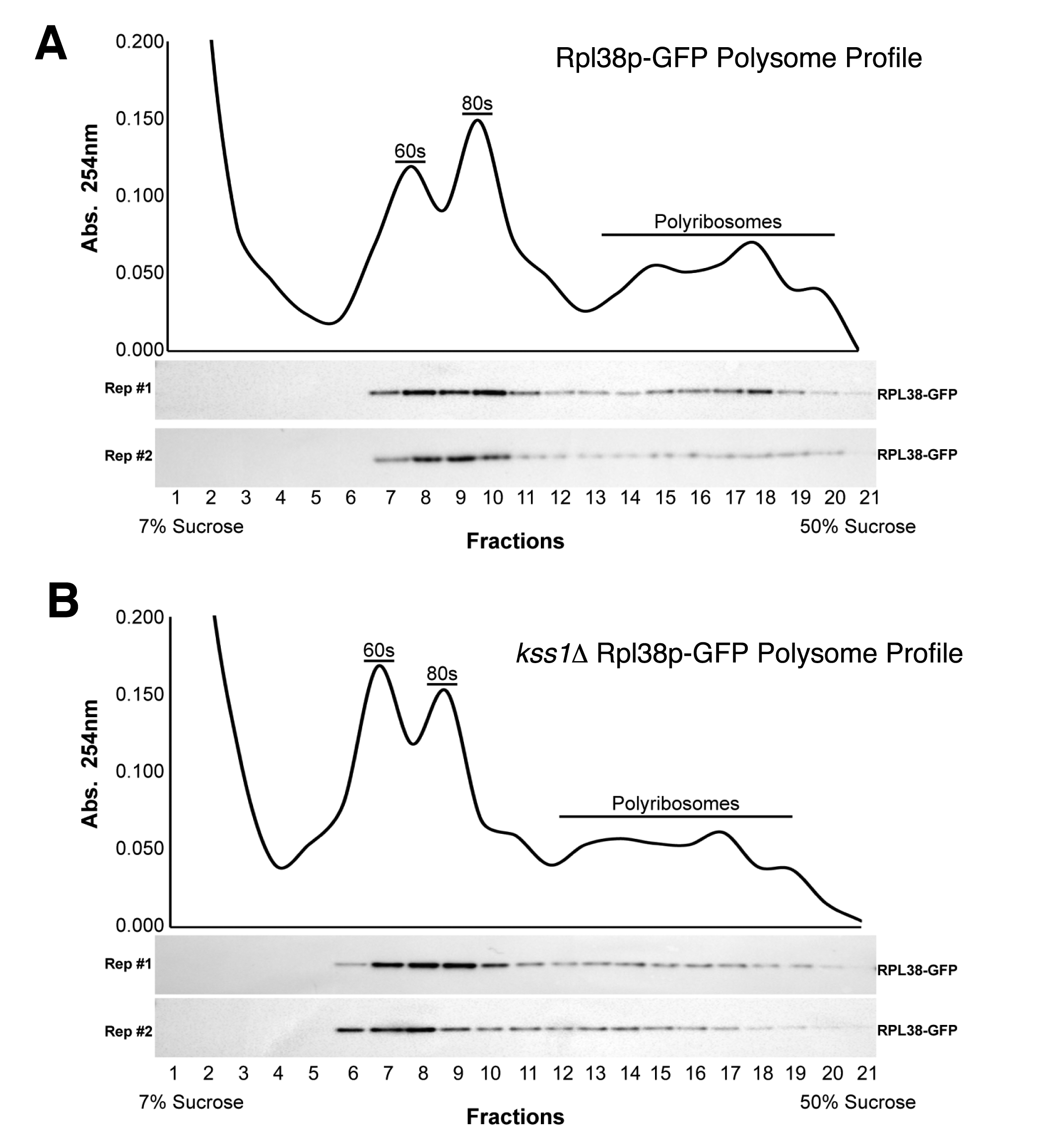

Supplement: S4 Fig — A) Analysis of a yeast strain with RPL38-GFP. Yeast cells were grown to log phase prior to the addition of cycloheximide at a final concentration of 0.1 mg/ml. The harvested cell pellets were resuspended in a slurry of 20 mM HEPES, 1.2% PVP40, 0.1 mg/ml cycloheximide, and Roche EDTA-free protease inhibitor cocktail prior to freezing in liquid nitrogen. Cell extracts were prepared from the frozen cells using a planetary ball mill under cryogenic conditions. The extracts were dissolved in polysome extraction buffer (20 mM HEPES, 140 mM KCl, 5 mM MgCl2, 0.1 mg/ml cycloheximide, 0.5 mM DTT, and protease inhibitor cocktail). The resulting lysates were centrifuged, and clarified lysates containing equivalent amounts of total RNA from treated cells were layered onto 12-ml continuous linear 7–50% (w/v) sucrose gradients in polysome buffer. Velocity sedimentation was performed by centrifuging the gradients at 35,000 rpm for 4 hours at 4 degrees C in a SW41 rotor. 500 μl fractions were manually collected from the top of the gradient to the bottom. Absorbance at 254 nm was measured for each fraction, and the fractions were analyzed for the presence of Rpl38p-GFP by Western blotting. Proteins were detected using Millipore Luminata Crescendo Western HRP Substrate and a BioRad ChemiDoc XRS imaging system with Image Lab software. In the polysome trace, peaks representing association of the large ribosomal subunit and monosome with mRNA are labeled 60S and 80S respectively. The heavier fractions containing polyribosomes are also identified. The polysome trace is from a single experiment representative of two biological replicates. Western blots of both biological replicates are located under the polysome trace. B) Analysis of the kss1Δ RPL38-GFP strain was carried out as described above. (TIF) [file pgen.1005564.s004.tif]

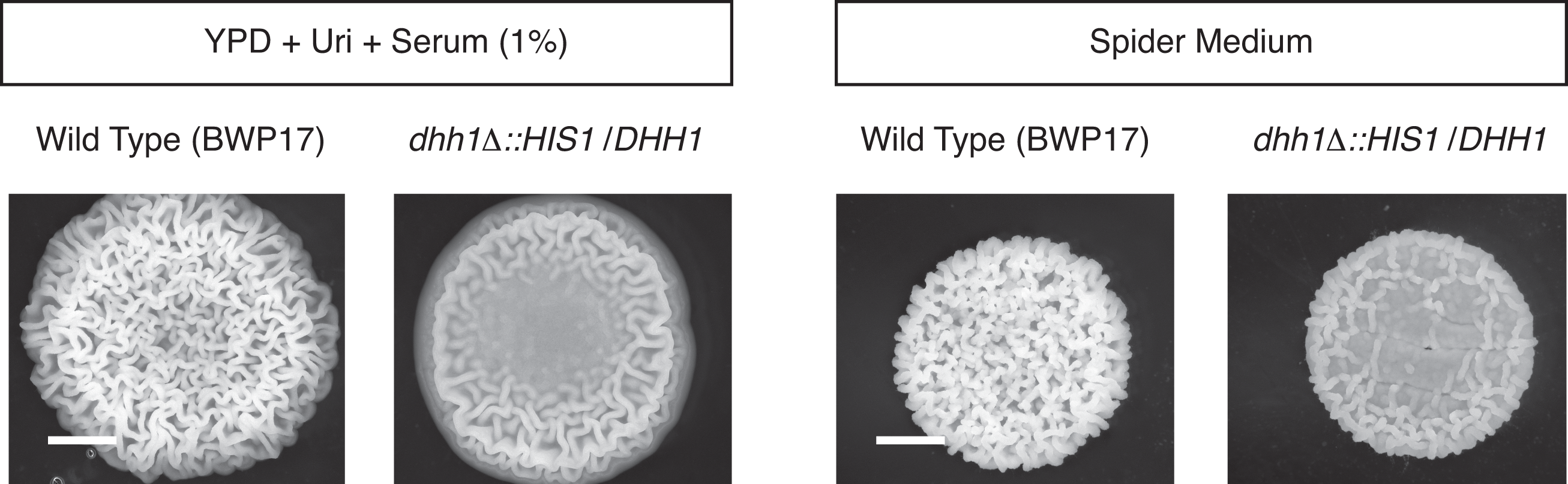

Supplement: S5 Fig — Images were obtained on indicated media after two days growth. Scale bar, 1 mm. (TIF) [file pgen.1005564.s005.tif]
