# Supplementary material for: Large-Scale Analysis of Kinase Signaling in Yeast Pseudohyphal Development Identifies Regulation of Ribonucleoprotein Granules
Source: PLoS Genet. 2015 Oct 8;11(10):e1005564. doi: 10.1371/journal.pgen.1005564 (PMC4598065; doi:10.1371/journal.pgen.1005564)
Supplement: S1 Table — (DOCX) [file pgen.1005564.s006.docx]

**Table S1.** Pseudohyphal growth kinase-dead phenotypes

| Kinase allele^a^ | Kinase pathway/family | Filamentous growth phenotype |
| --- | --- | --- |
| *ste20*-K649R | PAK | Decreased surface filamentation |
| *ste11*-K444R | MAPKKK | Decreased surface filamentation |
| *ste7*-K220R | MAPKK | Decreased surface filamentation |
| *kss1-*K42R | MAPK | Complex colony morphology |
| *fus3*-K42R | MAPK | Decreased invasive growth |
| *tpk2*-K99R | PKA | Decreased surface filamentation |
| *snf1*-K84R | AMP-activated kinase | Decreased surface filamentation |
| *elm1*-K117R | S/T Kinase regulating septins | Increased surface filamentation |

^a^.Kinase-dead mutants are diploid except for *kss1*-K42R and *fus3*-K42R
