# Supplementary material for: Large-Scale Analysis of Kinase Signaling in Yeast Pseudohyphal Development Identifies Regulation of Ribonucleoprotein Granules
Source: PLoS Genet. 2015 Oct 8;11(10):e1005564. doi: 10.1371/journal.pgen.1005564 (PMC4598065; doi:10.1371/journal.pgen.1005564)
Supplement: S6 Table — (DOCX) [file pgen.1005564.s011.docx]

**Table S6.** Strains used in this study

| Strain | Genotype | Source |
| --- | --- | --- |
| Y825 | *ura3-52 leu2*Δ*0 MAT***a** | M. Snyder (Stanford, CA) |
| HLY337 | *ura3-52 trp1-1 MAT*α | G. Fink (MIT, MA) |
| BWP17 | *ura3::*Δ*imm434/ura3::*Δ*imm434 his1::hisG/his1::hisG arg4::hisG/arg4::hisG* | FGSC (UMKC, MO) |
| yCAS1 | *ura3-52/ura3-52 leu2*Δ*0/leu2*Δ*0 MAT***a***/MAT*α pRS426 | This study |
| yCAS2 | *ura3-52/ura3-52 leu2*Δ*0/leu2*Δ*0 MAT***a***/MAT*α pRS416 | This study |
| yCAS3 | *ura3-52 leu2*Δ*0 arg4*Δ *lys1*Δ *MAT***a** | This study |
| yCD1 | *ura3-52 leu2*Δ*0 arg4*Δ *lys1*Δ *elm1*Δ *MAT***a** | This study |
| yCD2 | *ura3-52 leu2*Δ*0 arg4*Δ *lys1*Δ *fus3*Δ *MAT***a** | This study |
| yCD3 | *ura3-52 leu2*Δ*0 arg4*Δ *lys1*Δ *kss1*Δ *MAT***a** | This study |
| yCD4 | *ura3-52 leu2*Δ*0 arg4*Δ *lys1*Δ *ste7*Δ *MAT***a** | This study |
| yCD5 | *ura3-52 leu2*Δ*0 arg4*Δ *lys1*Δ *ste11*Δ *MAT***a** | This study |
| yCD6 | *ura3-52 leu2*Δ*0 arg4*Δ *lys1*Δ *ste20*Δ *MAT***a** | This study |
| yCD7 | *ura3-52 leu2*Δ*0 arg4*Δ *lys1*Δ *snf1*Δ *MAT***a** | This study |
| yCD8 | *ura3-52 leu2*Δ*0 arg4*Δ *lys1*Δ *tpk2*Δ *MAT***a** | This study |
| yTX1 | *ura3-52 leu2*Δ*0 arg4*Δ *lys1*Δ *elm1*Δ *MAT***a** + pDEST-*ELM1*-KD | This study |
| yTX2 | *ura3-52 leu2*Δ*0 arg4*Δ *lys1*Δ *fus3*Δ *MAT***a** + pDEST-*FUS3*-KD | This study |
| yTX3 | *ura3-52 leu2*Δ*0 arg4*Δ *lys1*Δ *kss1*Δ *MAT***a** + pDEST-*KSS1*-KD | This study |
| yTX4 | *ura3-52 leu2*Δ*0 arg4*Δ *lys1*Δ *snf1*Δ *MAT***a** + pDEST-*SNF1*-KD | This study |
| yTX5 | *ura3-52 leu2*Δ*0 arg4*Δ *lys1*Δ *ste7*Δ *MAT***a** + pDEST-*STE7*-KD | This study |
| yTX6 | *ura3-52 leu2*Δ*0 arg4*Δ *lys1*Δ *ste11*Δ *MAT***a** + pDEST-*STE11*-KD | This study |
| yTX7 | *ura3-52 leu2*Δ*0 arg4*Δ *lys1*Δ *ste20*Δ *MAT***a +** pDEST-*STE20*-KD | This study |
| yTX8 | *ura3-52 leu2*Δ*0 arg4*Δ *lys1*Δ *ste20*Δ *MAT***a** + pDEST-*TPK2*-KD | This study |
| yCAS4 | *ras2*Δ*::KanMX6*/*ras2*Δ*::KanMX6 ura3-52/ura3-52 leu2*Δ*0/LEU2 TRP1/trp1-1 MAT***a***/MATα* | This study |
| yCAS5 | *ras2*Δ*::KanMX6*/*ras2*Δ*::KanMX6 ura3-52/ura3-52 leu2*Δ*0/LEU2 TRP1/trp1-1 MAT***a***/MATα* + pRS416-*RAS2-*Y165F/T166A | This study |
| yKN1 | *flo8-*S3A *ura3-52 leu2*Δ*0 MAT***a** | This study |
| yCAS7 | *flo8*Δ*::KanMX6 ura3-52 leu2*Δ*0 MAT***a** + pFRE-*lacZ* | This study |
| yKN2 | *flo8-*S3A *ura3-52 leu2*Δ*0 MAT***a +** pFRE-*lacZ* | This study |
| yCAS923 | *flo8-*S3A-GFP-*KanMX6 ura3-52 leu2*Δ*0 MAT***a** | This study |
| yCAS101 | *KSS1-*GFP-*KanMX6 ura3-52 trp1-1 MAT*α | This study |
| yCAS102 | *FUS3-*GFP-*KanMX6 ura3-52 trp1-1 MAT*α | This study |
| yCAS103 | *STE20-*GFP-*KanMX6 ura3-52 trp1-1 MAT*α | This study |
| yCAS840 | *TPK2-*GFP-*TRP1* *ura3-52 trp1-1 MAT*α | This study |
| yCAS855 | *TPK2-*GFP-*TRP1* *IGO1-*mCherry-*KanMX6* *ura3-52 trp1-1 MAT*α | This study |
| yCAS757 | *FUS3-*GFP-*KanMX6* *IGO1-*mCherry-*HphMX4* *ura3-52 trp1-1 MAT*α | This study |
| yCAS758 | *KSS1-*GFP-*KanMX6* *IGO1-*mCherry-*HphMX4* *ura3-52 trp1-1 MAT*α | This study |
| yCAS759 | *STE20-*GFP-*KanMX6* *IGO1-*mCherry-*HphMX4* *ura3-52 trp1-1 MAT*α | This study |
| yCAS819 | *igo1*Δ*::KanMX6*/*igo1*Δ*::KanMX6 igo2::URA3/igo2::URA3 ura3-52/ura3-52 leu2_0/LEU2 TRP1/trp1-1 MAT***a***/MAT*α | This study |
| yCAS909 | *igo1*Δ*::KanMX6* *igo2*Δ*::URA3* *TPK2-*GFP-*TRP1 ura3-52 trp1-1 MAT*α | This study |
| yCAS794 | *ura3-52/ura3-52 leu2*Δ*0/LEU2 TRP1/trp1-1 igo1*Δ*::KanMX6*/*igo1*Δ*::KanMX6 MAT***a***/MATα* | This study |
| yCAS255 | *ura3-52/ura3-52 leu2*Δ*0/LEU2 TRP1/trp1-1 dhh1*Δ*::KanMX6*/*dhh1*Δ*::KanMX6 MAT***a***/MATα* | This study |
| yCAS682 | *ura3-52/ura3-52 leu2*Δ*0/LEU2 TRP1/trp1-1 dhh1*Δ*::KanMX6*/*dhh1*Δ*::KanMX6 MAT***a***/MATα* | This study |
| yCAS176 | *ura3-52/ura3-52 leu2*Δ*0/LEU2 TRP1/trp1-1 pat1*Δ*::KanMX6*/*pat1*Δ*::KanMX6 MAT***a***/MATα* | This study |
| yCAS177 | *ura3-52/ura3-52 leu2*Δ*0/LEU2 TRP1/trp1-1 pbp1*Δ*::KanMX6*/*pbp1*Δ*::KanMX6 MAT***a***/MATα* | This study |
| yCAS175 | *ura3-52/ura3-52 leu2*Δ*0/LEU2 TRP1/trp1-1 sbp1*Δ*::KanMX6*/*sbp1*Δ*::KanMX6 MAT***a***/MATα* | This study |
| yCAS452 | *ste12*Δ*::KanMX6 ura3-52 trp1-1 MAT*α + pFRE-*lacZ* | This study |
| yCAS810 | *ccr4*Δ*::KanMX6 ura3-52 trp1-1 MAT*α + pFRE-*lacZ* | This study |
| yCAS454 | *dhh1*Δ*::KanMX6 ura3-52 trp1-1 MAT*α + pFRE-*lacZ* | This study |
| yCAS941 | *lsm1*Δ*::KanMX6 ura3-52 trp1-1 MAT*α + pFRE-*lacZ* | This study |
| yCAS943 | *pat1*Δ*::KanMX6 ura3-52 trp1-1 MAT*α + pFRE-*lacZ* | This study |
| yCAS537 | *pbp1*Δ*::KanMX6 ura3-52 trp1-1 MAT*α + pFRE-*lacZ* | This study |
| yCAS538 | *sbp1*Δ*::KanMX6 ura3-52 trp1-1 MAT*α + pFRE-*lacZ* | This study |
| yCAS540 | *xrn1*Δ*::KanMX6 ura3-52 trp1-1 MAT*α + pFRE-*lacZ* | This study |
| yCAS969 | *ura3-52/ura3-52 leu2*Δ*0/LEU2 TRP1/trp1-1 lsm1*Δ*::KanMX6*/*lsm1*Δ*::KanMX6* *MAT***a***/MATα* + *STE11-4-*pRS416 | This study |
| yCAS971 | *ura3-52/ura3-52 leu2*Δ*0/LEU2 TRP1/trp1-1 pat1*Δ*::KanMX6*/*pat1*Δ*::KanMX6* *MAT***a***/MATα* + *STE11-4-*pRS416 | This study |
| yCAS968 | *ura3-52/ura3-52 leu2*Δ*0/LEU2 TRP1/trp1-1 lsm1*Δ*::KanMX6*/*lsm1*Δ*::KanMX6* *MAT***a***/MATα* + *STE7-*S368P-pRS416 | This study |
| yCAS970 | *ura3-52/ura3-52 leu2*Δ*0/LEU2 TRP1/trp1-1 pat1*Δ*::KanMX6*/*pat1*Δ*::KanMX6* *MAT***a***/MATα* +  *STE7-*S368P-pRS416 | This study |
| yCAS636 | *ura3-52 leu2*Δ*0 FUS3*-mCherry-*KanMX6 MAT***a** | This study |
| yCAS637 | *ura3-52 leu2*Δ*0 KSS1*-mCherry-*KanMX6 MAT***a** | This study |
| yCAS635 | *ura3-52 leu2*Δ*0 STE20*-mCherry-*KanMX6 MAT***a** | This study |
| yCAS688 | *ura3-52 leu2*Δ*0 TPK2*-mCherry-*KanMX6 MAT***a** | This study |
| yKN1 | *ura3-52 leu2*Δ*0 FUS3*-mCherry-*KanMX6 MAT***a** + pRP1194 + pPS2037 | This study |
| yKN2 | *ura3-52 leu2*Δ*0 KSS1*-mCherry-*KanMX6 MAT***a** + pRP1194 + pPS2037 | This study |
| yKN3 | *ura3-52 leu2*Δ*0 STE20*-mCherry-*KanMX6 MAT***a** + pRP1194 + pPS2037 | This study |
| yKN4 | *ura3-52 leu2*Δ*0 TPK2*-mCherry-*KanMX6 MAT***a** + pRP1194 + pPS2037 | This study |
| yKN5 | *ura3-52 leu2*Δ*0 kss1*Δ*::KanMX6 MAT***a** + pRP1194 + pPS2037 | This study |
| yKN6 | *ura3-52 leu2*Δ*0 FUS3*-GFP-*KanMX6 MAT***a** + pRP1194 + pDS7 | This study |
| yKN7 | *ura3-52 leu2*Δ*0 KSS1*-GFP-*KanMX6 MAT***a** + pRP1194 + pDS7 | This study |
| yKN8 | *ura3-52 leu2*Δ*0 STE20*-GFP-*KanMX6 MAT***a** + pRP1194 + pDS7 | This study |
| yKN9 | *ura3-52 leu2*Δ*0 TPK2*-GFP-*KanMX6 MAT***a** + pRP1194 + pDS7 | This study |
| yKN11 | *dhh1*Δ*::CdHIS1/DHH1 ura3::*Δ*imm434/ura3::*Δ*imm434 his1::hisG/his1::hisG arg4::hisG/arg4::hisG* | This Study |
