# Supplementary material for: Large-Scale Analysis of Kinase Signaling in Yeast Pseudohyphal Development Identifies Regulation of Ribonucleoprotein Granules
Source: PLoS Genet. 2015 Oct 8;11(10):e1005564. doi: 10.1371/journal.pgen.1005564 (PMC4598065; doi:10.1371/journal.pgen.1005564)
Supplement: S7 Table — (DOCX) [file pgen.1005564.s012.docx]

**Table S7.** Plasmids used in this study

| Plasmid | Description | Source |
| --- | --- | --- |
| pFRE-LacZ | P_FRE(TEC1)_::*lacZ*, *URA3*, 2μ, Amp^r^ | Madhani and Fink, 1997 |
| pRS415 | *LEU2*, Cen, Amp^r^ | Sikorski and Hieter, 1989 |
| pRS416 | *URA3*, Cen, Amp^r^ | Sikorski and Hieter, 1989 |
| p426GPD | P_GPD3_, *URA3*, 2μ, Amp^r^ | Mumberg *et al*, 1995 |
| pRS416-*ELM1* | P_ELM1_-*ELM1*, *URA3*, Cen, Amp^r^ | This study |
| pRS416-*FUS3* | P_FUS3_-*FUS3*, *URA3*, Cen, Amp^r^ | This study |
| pRS416-*KSS1* | P_KSS1_-*KSS1*, *URA3*, Cen, Amp^r^ | This study |
| pRS416-*SNF1* | P_SNF1_-*SNF1*, *URA3*, Cen, Amp^r^ | This study |
| pRS416-*STE7* | P_STE7_-*STE7*, *URA3*, Cen, Amp^r^ | This study |
| pRS416-*STE11* | P_STE11_-*STE11*, *URA3*, Cen, Amp^r^ | This study |
| pRS416-*STE20* | P_STE20_-*STE20*, *URA3*, Cen, Amp^r^ | This study |
| pRS416-*TPK2* | P_TPK2_-*TPK2*, *URA3*, Cen, Amp^r^ | This study |
| pRS416-*ELM1-*K117R | P_ELM1_-*ELM1-*K117R, *URA3*, Cen, Amp^r^ | This study |
| pRS416-*FUS3-*K42R | P_FUS3_-*FUS3-*K42R, *URA3*, Cen, Amp^r^ | This study |
| pRS416-*KSS1*-K42R | P_KSS1_-*KSS1-*K42R, *URA3*, Cen, Amp^r^ | This study |
| pRS416-*SNF1*-K84R | P_SNF1_-*SNF1-*K84R, *URA3*, Cen, Amp^r^ | This study |
| pRS416-*STE7*-K220R | P_STE7_-*STE7-*K220R, *URA3*, Cen, Amp^r^ | This study |
| pRS416-*STE11*-K444R | P_STE11_-*STE11-*K444R, *URA3*, Cen, Amp^r^ | This study |
| pRS416-*STE20*-K649R | P_STE20_-*STE20-*K649R, *URA3*, Cen, Amp^r^ | This study |
| pRS416-*TPK2*-K99R | P_TPK2_-*TPK2-*K99R, *URA3*, Cen, Amp^r^ | This study |
| pDEST-*ELM1*-KD | P_ELM1_-*ELM1-*K117R-HA, *LEU2*, Cen, Amp^r^ | This study |
| pDEST-*FUS3*-KD | P_FUS3_-*FUS3-*K42R-HA, *LEU2*, Cen, Amp^r^ | This study |
| pDEST-*KSS1*-KD | P_KSS1_-*KSS1-*K42R-HA, *LEU2*, Cen, Amp^r^ | This study |
| pDEST-*SNF1*-KD | P_SNF1_-*SNF1-*K84R-HA, *LEU2*, Cen, Amp^r^ | This study |
| pDEST-*STE7*-KD | P_STE7_-*STE7-*K220R-HA, *LEU2*, Cen, Amp^r^ | This study |
| pDEST-*STE11*-KD | P_STE11_-*STE11-*K444R-HA, *LEU2*, Cen, Amp^r^ | This study |
| pDEST-*STE20*-KD | P_STE20_-*STE20-*K649R-HA, *LEU2*, Cen, Amp^r^ | This study |
| pDEST-*TPK2*-KD | P_TPK2_-*TPK2-*K99R-HA, *LEU2*, Cen, Amp^r^ | This study |
| p*RAS2-*Y165F/T166A | P_RAS2_-*RAS2-*Y165F/T166A, *URA3*, Cen, Amp^r^ | This study |
| pRS415-*STE11-4* | P_STE11_-*STE11-4* *LEU2*, Cen, Amp^r^ | This study |
| pRS416-*STE7-*S368P | P_STE11_-*STE7-*S368P *URA3*, Cen, Amp^r^ | This study |
| pRP1194 | P_GPD3_-U1A-GFP, *LEU2*, Amp^r^ | Brengues *et al*, 2005 |
| pDS7 | P_GPD3_-U1A-mCherry, *LEU2*, Amp^r^ | This study |
| pPS2037 | P_PGK1_-*PGK1-*16x U1A binding sites, *URA3*, Amp^r^ | Brodsky and Silver, 2000 |
